# Disruption of MCL1/BOK transmembrane interaction as a novel strategy to induce cell death in tumours

**DOI:** 10.1038/s41419-026-09074-5

**Published:** 2026-07-03

**Authors:** M. Buffa, D. Leiva, A. Garcia-Jareño, M. Garcés-Flores, E. Díaz, F. Lucantoni, M. Telechea, L. Chen, E. Lucendo, R. Shalaby, F. Lolicato, W. Kulig, AJ. Garcia-Saez, G. Moreno-Bueno, M. Sancho, M. Orzáez

**Affiliations:** 1 Centro de Investigación Príncipe Felipe, Targeted Therapies on Cancer and Inflammation Laboratory, Valencia, Spain; 2 PhD in Chemistry Program, Universitat Politècnica de València, Valencia, Spain; 3 Fundación MD Anderson Internacional, Madrid, Spain; 4 Translational Cancer Research Group, Area 3 Cancer, Instituto Ramón y Cajal de Investigación Sanitaria (IRYCIS), Madrid, Spain; 5 PhD in Biotechnology Program, Universitat Politècnica de València, Barcelona, Spain; 6 Institute of Genetics, University of Cologne, Cologne, Germany; 7 Max Planck Institute of Biophysics, Frankfurt am Main, Germany; 8 Heidelberg University Biochemistry Center, Heidelberg, Germany; 9 Department of Physics, University of Helsinki, Helsinki, Finland; 10 Centro de Investigación Biomédica en Red, Área Cáncer, CIBERONC, Instituto de Salud Carlos III, Madrid, Spain; 11 Department of Biochemistry and Molecular Biology, Faculty of Pharmacy, University of Valencia, Burjassot, Spain

**Keywords:** Breast cancer, High-throughput screening

## Abstract

MCL1, an anti-apoptotic BCL-2 family member, is frequently overexpressed in multiple tumour types and correlates with poor prognosis; however, targeting its cytosolic domain to release pro-apoptotic effectors has been limited by cardiotoxicity in clinical trials. In this study, we describe a chemically mediated disruption of the MCL1/BOK transmembrane interaction that induces tumour cell death without affecting viability in 3D cardiomyocyte cultures. Molecular dynamics simulations and LUV-based assays converged to show that the MBoIN179 disrupts transmembrane dimerization, thereby restoring BOK pore formation inhibited by MCL1. In cells, interference with the MCL1/BOK transmembrane interaction promoted relocalization of BOK from the endoplasmic reticulum to mitochondria, where it engaged a BOK-dependent cell death program in both 2D and 3D breast cancer models, leading to reduced tumour growth and metastasis in vivo. Analysis of patient tumour microarrays further showed that BOK is overexpressed in aggressive breast cancer subtypes and correlates with poor prognosis. Together, these findings identify the MCL1/BOK transmembrane interaction as a tumour-selective vulnerability that can be engaged to promote antitumour responses with cardiac safety, highlighting transmembrane interactions as viable molecular targets.

## Introduction

MCL1 is an anti-apoptotic member of the BCL-2 (B-cell lymphoma protein 2) family [1]. Gene amplification of MCL1 (myeloid cell leukemia-1) occurs in approximately 10% of tumours and is associated with poor prognosis and reduced treatment responses [2–7]. The important role of MCL1 in tumours has led to the development of several small-molecule inhibitors, some of which have reached clinical trials due to their efficacy in preclinical models. The mechanism of action of these inhibitors is based on mimicking the interaction of MCL1 with the cytosolic BH3-binding domains of pro-apoptotic proteins such as NOXA, BIM or BAK, leading to the release of these effector proteins and the subsequent induction of apoptosis through BAX/BAK-mediated mitochondria pore formation. Unfortunately, many of these compounds were discontinued because of cardiotoxicity observed in patients. This toxicity reflects that human cardiomyocytes also rely on MCL1/BAK interaction for their survival, providing a mechanistic explanation for the on-target cardiotoxicity observed in patients [8, 9]. Furthermore, the efficacy of these inhibitors could also be limited by the resistance of pro-apoptotic proteins such as BIM and PUMA to BH3-mimetic displacement, due to a “double-bolt lock” mechanism involving both their BH3 domain and carboxyl-terminal sequence [10, 11]. Thus, there is an urgent need to develop new strategies that counteract MCL1 overexpression in tumours without compromising cardiomyocyte viability [12–16].

Recent work, including our own, has shown that MCL1 interacts with BOK through the transmembrane domain (TMD) [17–20]. BOK is a protein homologous to BAX and BAK, that mainly resides in the endoplasmic reticulum (ER) but is also capable of forming pores in the mitochondrial membrane [21, 22]. It has been implicated as a pro-apoptotic factor during ER stress–induced liver injury [23] and can induce mitochondrial outer membrane permeabilization independently of BAX and BAK [24]. Notably, BOK expression in cardiomyocytes is low, suggesting a potential therapeutic window [25].

In previous studies, we demonstrated that overexpression of the MCL1 TMD disrupts the MCL1/BOK interaction, leading to the release of BOK, suggesting that targeting this interaction may represent a viable approach to induce BOK-dependent cell death in tumour cells. [17] Although the highly hydrophobic and poorly characterized membrane environment presents a significant challenge for chemical intervention, here we explore targeting the MCL1/BOK TMD interaction as an alternative approach to induce tumour cell death.

## Results

### Identification of MCL1/BOK transmembrane interaction disruptors

Disruption of the heterotypic interaction between MCL1 and BOK TMDs may constitute a promising strategy to liberate BOK and trigger apoptosis in tumour cells. To further explore this hypothesis, we developed an image-based bimolecular fluorescence complementation (BiFC) screening platform to identify and validate chemical inhibitors capable of selectively disrupting the MCL1/BOK TMD interaction.

**Fig. 1:**
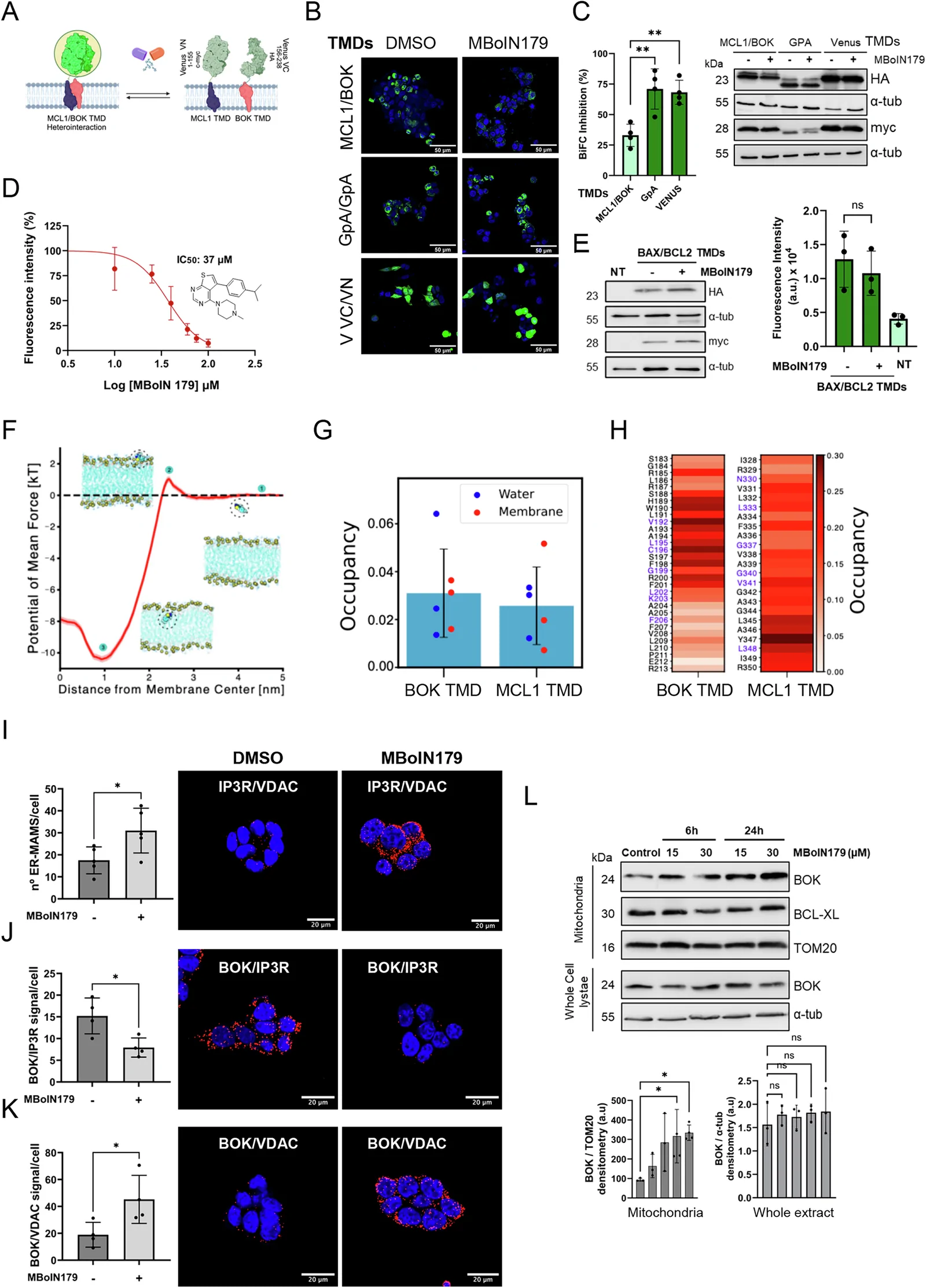
MBoIN179 disrupts MCL1/BOK TMDs complexes in live cells. **A** Schematic view of the rationale of the HTS assay. **B** Oligomerization analysis of the indicated TMDs constructs measured by BiFC in HCT116 cells in the presence of MBoIN179 (50 µM). The Left panel shows representative confocal images of the BiFC assay. Hetero-oligomerization of MCL1/BOK TMDs, homo-oligomerization of GpA TMDs as a nonrelated TMD, and Venus (V VC/VN) reconstitution without TM interaction were evaluated. Scale bar corresponds to 50 µm. **C** Percentage of BiFC signal inhibition upon MBoIN179 treatment. Graph represents the mean ± SD, *n* = 4. Data were analyzed using one-way ANOVA followed by Tukey’s post-hoc test; **p < 0.01 indicates a significant difference between MCL1/BOK TMDs and other non-related TMDs. The right panel illustrates protein expression level of the constructs: HA epitope immunoblotting corresponds to VC- TMD expression and myc epitope blotting to VN- TMD. α-tubulin was used as a loading control. Quantification of 3 replicates is shown in Fig. S1J. **D** Dose-response activity of MBoIN179 in BiFC hetero-interaction assay. HCT116 cells transfected with VN MCL1 TMD and VC BOK TMD and treated with MBoIN179 at 10, 25, 40, 60, 75 and 100 µM for 24 h. Data are presented as mean ± SD of five independent experiments. **E** Hetero-oligomerization of BAX/BCL2 TMDs evaluated by BiFC. The right panel illustrates the BiFC signal upon MBoIN179 treatment. Graph represents the mean ± SD, *n* = 3. Data were analyzed using one-way ANOVA followed by Tukey’s post-hoc test. The left panel corresponds to the protein expression level of the constructs analyzed by Western blot. Quantification is included in Fig. S1K. **F** Potential of mean force (PMF) for MBolN179 along the membrane normal in a POPC bilayer, obtained from umbrella-sampling simulations. Shaded regions represent statistical uncertainty estimated from 200 bootstrap iterations. **G** Contact occupancy of MBolN179 near the transmembrane (TM) helices of BOK and MCL1 in unbiased simulations. Blue and red points indicate simulations initiated with MBoIN179 in water or pre-inserted in the membrane, respectively. **H** Residue-level contact frequencies between MBolN179 and the TM helices of BOK (left) and MCL1 (right), calculated from frames in which the compound is bound. Contacts were identified whenever an atom of MBolN179 is within 0.6 nm of any protein atom. Residues highlighted in blue were previously mapped to the BOK–MCL1 interface [17]. **I** Representative confocal images and quantification of in situ PLA targeting VDAC1 and IP3R interactions in HCT116 cells treated with MBoIN179 (50 µM) for 24 h. **J** Representative confocal images and quantification of in situ PLA targeting BOK and IP3R interactions in HCT116 cells treated with MBoIN179 (50 µM) for 24 h. **K** Representative confocal images and quantification of in situ PLA targeting BOK and VDAC interactions in HCT116 cells treated with MBoIN179 (50 µM) for 24 h. In all three cases, the experiments were performed at least four times; the graph represents the mean ± SD; Student’s t-test was performed with *p < 0.05. Scale bar 20 µm. **L** Detection of BCLXL, BOK and the mitochondrial marker TOM20 expression in HCC1954 cells after 6 and 24 h of MBoIN179 treatment at the indicated concentrations in the mitochondrial fraction and also, BOK and α-tubulin in the whole cell extract. Quantifications of immunoblotting are shown in the lower panels. The graph represents mean ± SD of three different experiments. One-way ANOVA followed by Dunnett’s multiple comparisons test revealed significant differences compared to the control group in mitochondrial fractions; *p < 0.05. One-way ANOVA followed by Dunnett’s multiple comparisons test revealed significant differences compared to the control group in mitochondrial fractions; *p < 0.05.

HCT116 human colorectal carcinoma cells co-expressing the TMDs of MCL1 and BOK, fused respectively to the N- and C-terminal fragments of the Venus fluorescent protein, were employed in a high-throughput screening (HTS) of the MyriaScreen small-molecule library (Sigma-Aldrich). In this BiFC system, interaction between MCL1 and BOK TMDs leads to reconstitution of Venus fluorescence [17, 26], whereas disruption of this interaction results in a reduction in fluorescence intensity (Figs. 1A and S1A–E). Hits identified in a primary HTS, as inhibitors of the MCL1 and BOK TMD hetero-interaction, were evaluated in a secondary screening to exclude those compounds either interfering with Venus reconstitution or interfering with the dimerization of an unrelated TMD, the glycophorin A (GpA) TMD, a well-known transmembrane dimer [27, 28] (Fig. S1F–H). These analyses allowed us to select MBoIN179 (Fig. S1I) as a molecule that does not significantly interfere with GpA and Venus oligomerization and is able to selectively disrupt MCL1 and BOK TMDs heterodimer formation (Fig. 1B). The expression of the different constructs was not affected by the presence of the molecule (Figs. 1C and S1J). Treatment with MBoIN179 disrupts the MCL1/BOK TMD interaction in a dose-dependent manner, with an IC_50_ in the micromolar range (Fig. 1D). We also evaluated the effect of MBoIN179 on BCL-2/BAX transmembrane interactions and observed no detectable impact (Figs. 1E and S1K), supporting the specificity of its action and indicating that it does not broadly perturb TMD interactions within the BCL-2 family. In contrast, BOK homooligomerization was reduced in the presence of the compound (Fig. S1L). This effect is consistent with a partial overlap of residues mediating BOK homotypic and MCL1/BOK heterotypic transmembrane interactions [17], suggesting that disruption may also facilitate BOK release and promote its participation in proteolipidic pore formation [22].

### Molecular dynamics analysis of MBolN179 membrane partitioning and MCL1/BOK transmembrane interactions

To better understand MBoIN179’s behaviour in a membrane environment, we performed atomistic molecular dynamics (MD) simulations in 1-Palmitoyl-2-oleoyl-sn-glycero-3-phosphocholine (POPC) lipid bilayers, complemented by free-energy calculations. As a first step, we assessed the compound’s membrane permeability to quantify its preferred localization within the bilayer. To this end, we employed biased MD simulations using the umbrella-sampling technique [29, 30] to compute the potential of mean force (PMF) along the membrane normal and thereby estimate the energetically favourable region for the compound. As shown in Fig. 1F, MBoIN179 strongly favours membrane partitioning. The PMF displays a pronounced minimum of 10.4 k_B_T at a depth of 0.9 nm from the bilayer center, indicating that MBolN179 preferentially resides within the hydrophobic interior of the POPC membrane, near the region of the lipid phosphate groups. The free-energy barrier associated with moving from bulk water into the membrane is low ( ≈ 1 k_B_T), consistent with spontaneous insertion from the aqueous phase. Overall, the free-energy profile clearly demonstrates that the equilibrium population of MBolN179 is shifted toward the membrane interior. Next, to investigate how MBolN179 interacts with the TMD helices of both MCL1 and BOK, we performed unbiased MD simulations using a 1:1 protein–compound ratio. We prepared two types of systems: one in which MBolN179 started in the aqueous phase, and another in which it was pre-inserted into the membrane. Each setup was simulated in triplicate, with randomized initial compound positions, for 500 ns per replicate to ensure independent sampling. As shown in Fig. 1G, regardless of whether MBoIN179 initially resided in water or in the membrane, its overall occupancy near the proteins was low, with only ~3% of all frames showing MBolN179 bound to MCL1 or BOK. However, analysis restricted to the bound frames revealed that MBolN179 forms persistent hydrophobic contacts with the TMD helices of both proteins (Fig. 1H). For BOK, the primary contact hotspots cluster along the mid-helix region (approximately S188–F201), whereas for MCL1 they span the upper–central segment (approximately V338–I348) (Fig. S1M, N). Notably, many of the residues showing the highest contact frequencies overlap with positions previously identified in our earlier work as part of the BOK–MCL1 heterodimer interface [17]. This pattern suggests that MBolN179 tends to occupy the same membrane-embedded surface used for TMD–TMD association in the heterodimer, where it could sterically and/or energetically interfere with helix–helix packing.

### Chemical disruption of MCL1/BOK TMD interaction promotes transorganellar movement of BOK from the ER to the mitochondria

Recently, recombinant BOK has been shown to form pores in mitochondrial membranes. [22] However, given that BOK is predominantly localized in the endoplasmic reticulum (ER) [31], it remains unclear how endogenous BOK translocates to mitochondria to promote mitochondrial outer membrane permeabilization (MOMP).

Our previous studies demonstrated that MCL1 and BOK TMDs participate in endomembrane fusion events between the mitochondria and the ER [17]. To study if MBoIN179 promotes the movement of BOK between both organelles through these contact structures, we employed proximity ligation assays (PLAs) to visualize and quantify endogenous ER-mitochondria associated membranes (MAMs) [32, 33] with antibodies targeting VDAC1 and IP3R1 proteins from the outer mitochondrial and ER membranes, respectively (Fig. 1I). Quantification of MAMs confirmed that treatment with MBoIN179 produced an increase of inter-organelle contacts. To elucidate if this increase in MAMs correlates with a transorganellar movement of BOK from the ER to the mitochondria, we performed PLAs to study changes in the proximity between BOK and IP3R or BOK and VDAC as a consequence of treatment. Our results showed a decrease in IP3R/ BOK proximity (Fig. 1J) together with an increase in VDAC/BOK proximity (Fig. 1K), consistent with a movement of BOK from the ER to the mitochondria upon treatment. In agreement with these observations, BOK levels in the mitochondrial membrane fraction increased over time following treatment, whereas other BCL-2 proteins were unaffected (Fig. 1L). Our findings support that disruption of the MCL1 and BOK TMD interactions increases MAM formation.

### BOK-dependent membrane permeabilization is inhibited by MCL1 and rescued by MBoIN179

**Fig. 2:**
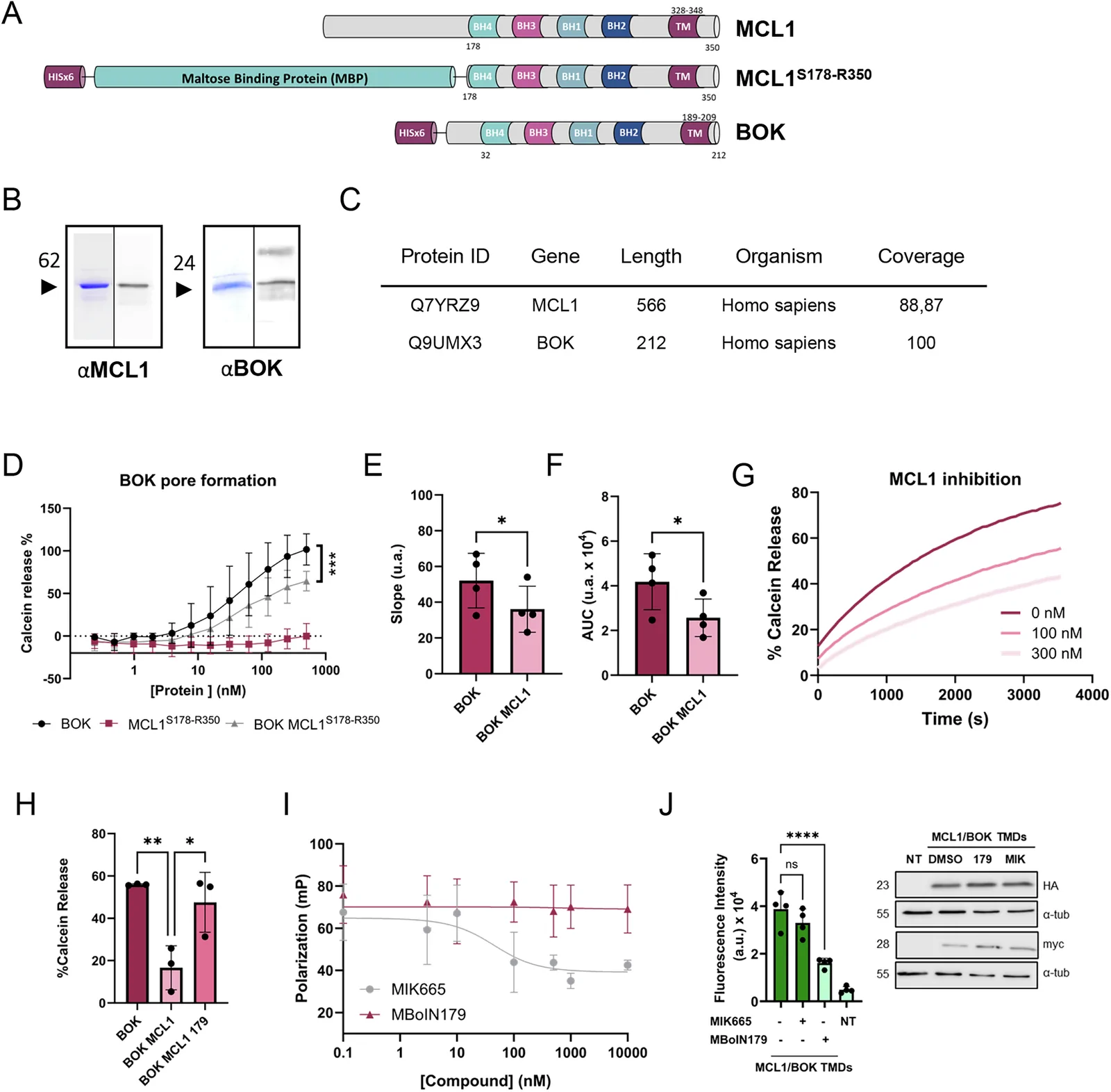
MCL1 inhibits BOK-mediated membrane permeabilization. **A** Schematic representation of the recombinant protein constructs used in the figure. **B** Coomassie blue staining and immunoblot analysis of purified recombinant BOK and MCL1^S178-R350^. **C** Protein identification by mass spectrometry. **D** Calcein-release assay in large unilamellar vesicles (LUVs) mimicking mitochondrial membranes showing that BOK induces pore formation, whereas MCL1^S178-R350^ alone has no effect. Co-incubation of MCL1^S178-R350^ with BOK suppresses BOK-mediated permeabilization. Protein concentrations ranged from 0.2 to 500 nM. Triton X-100–treated vesicles were set as 100% release, and untreated vesicles as 0%. Data are mean ± SD from *n* = 4 independent experiments. Statistical analysis was performed using two-way ANOVA with Sidak’s post-test; differences between BOK and BOK + MCL1^S178-R350^ were significant (***p < 0.001). **E** Comparison of initial slopes of calcein-release traces for BOK versus BOK + MCL1^S178-R350^. Data are mean ± SD from *n* = 4 independent experiments. Student’s t-test displayed *p < 0.05. **F** Area under the curve (AUC) analysis for the conditions shown in (**E**). Data are mean ± SD from *n* = 4 independent experiments. Student’s t-test displayed *p < 0.05. **G** Kinetic traces of calcein release from LUVs incubated with BOK (125 nM) in the presence of increasing concentrations of MCL1^S178−R350^ (0, 100, and 300 nM). Data are mean ± SD from *n* = 3 independent experiments. **H** Reversing MCL1^S178-R350^-mediated inhibition of BOK permeabilization at 125 nM of each protein by 50 nM MBoIN179. Data are mean ± SD from *n* = 3 independent experiments. One-way ANOVA followed by Dunnett’s multiple comparisons test revealed significant differences between BOK + MCL1^S178-R350^ and BOK + MCL1^S178-R350^ + MBoIN179, *p < 0.05. **I** MBolN179 does not displace the BAK BH3 peptide from MCL1. Carboxyfluorescein-labeled BAK BH3 peptide (CF-Bak) was incubated with MCL1ΔTM in the presence of increasing concentrations of MIK665 or MBolN179, and fluorescence polarization was measured. While MIK665 disrupts the interaction, decreasing the polarization signal (gray line), MBoIN179 does not affect the signal (purple line). Data points represent the mean ± SD, *n* = 4. **J** Hetero-oligomerization of MCL1/BOK TMDs evaluated by BiFC in the presence or absence of MIK665 (15 µM) or MBoIN179 (27 µM). Graph represents the mean ± SD, *n* = 4. Data were analyzed using one-way ANOVA followed by Tukey’s post-hoc test. The right panel corresponds to the protein expression level of the constructs analyzed by Western blot. Quantification is included in Fig. S2A.

To further investigate the mechanism of action of MBoIN179, we reconstituted an in vitro assay using an established mitochondrial model to recapitulate the inhibitory action of MCL1 over the pore-formation activity of BOK [34]. Large unilamellar vesicles (LUVs) loaded with calcein were used to analyse the behaviour of BOK and MCL1 in the membrane. Disruption of the integrity of the LUVs can be detected by the release of the fluorescent dye calcein. For the expression of recombinant BOK FL, we followed recently described protocols [22]. In the case of MCL1, a truncated version of the protein containing the C-terminal transmembrane region and fused at its N-terminus to the maltose binding protein (named from now MCL1^S178-R350^) was produced in E coli BL21 pLys DE3 (RIPL) (Fig. 2A). After purification, the identity of both proteins (BOK and MCL1^S178-R350^) was successfully corroborated both by immunoblotting and mass spectrometry analysis (Fig. 2B, C). Incubation of LUVs with different concentrations of MCL1^S178-R350^ did not compromise membrane structure. However, BOK induced dose-dependent membrane permeabilization, while preincubation of both BOK with MCL1^S178-R350^ significantly reduced calcein release (Fig. 2D, G). Both the permeabilization rate (slope; Fig. 2E) and the total calcein-release (area under the curve; Fig. 2F) are compromised in the presence of the anti-apoptotic protein MCL1 ^S178-R350^. To our knowledge, this provides the first *in*-*vitro* functional evidence that MCL1 counteracts the pore-forming activity of BOK. Then, we studied the effect of MBoIN179 treatment on the activity of these two proteins. Interestingly, the presence of MBoIN179 counteracts the inhibitory effect of MCL1^S178-R350^ (Fig. 2H), reinforcing MBoIN179’s mechanism of action as an inhibitor of MCL1/BOK TMD interaction. To discard that MBoIN179 was interfering with the BH3 binding domain of MCL1, we developed a fluorescence polarization assay where Carboxifluorescein labeled BH3 Bak peptide was incubated with MCL1 and increasing concentration of MIK665 or MBoIN179. Interestingly, while MIK665 produces the release of CF-Bak, decreasing polarization, no effect was observed upon incubation with MBoIN179 (Fig. 2I), further confirming its specificity towards the MCL1/BOK TMD interaction. Moreover, we evaluated the effect of MIK665 on the MCL1/BOK TMD interaction using the BiFC assay and demonstrated that this BH3 mimetic does not disrupt the transmembrane interaction (Fig. 2J), further supporting the distinct mechanisms of action of the two compounds.

### BOK is overexpressed in aggressive breast cancers

**Fig. 3:**
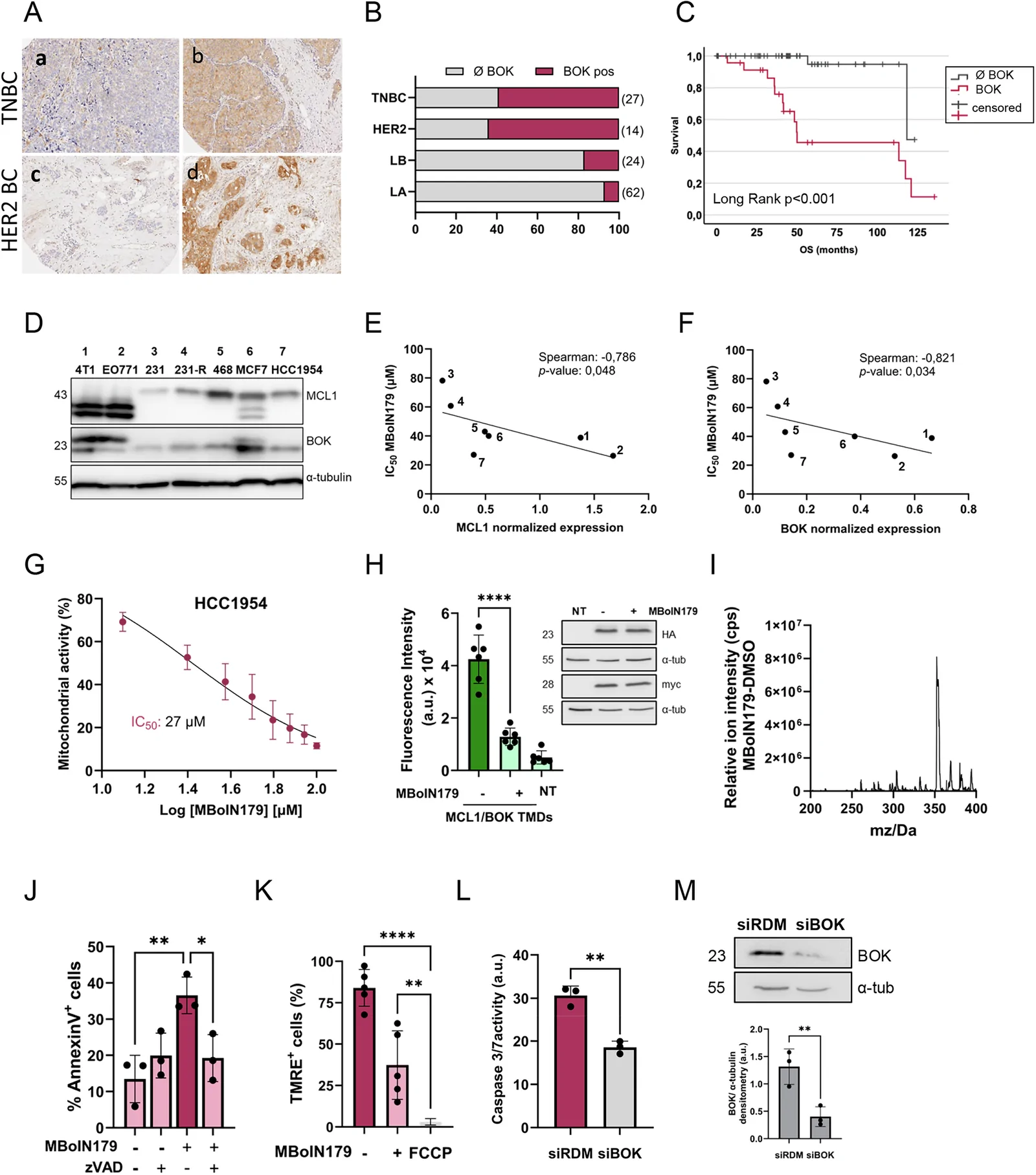
Role of BOK in breast cancer. **A** Representative images showing BOK expression in tumour samples from TMA: (a) TNBC with no detectable BOK expression, (b) TNBC with high BOK expression, (c) HER2-positive breast cancer with no BOK expression and (d) HER2-positive breast cancer with high BOK expression. Scale bar = 10x. **B** Distribution of BOK expression (positive vs. negative) across breast cancer phenotypes (Luminal A, Luminal B, HER2-enriched, and TNBC) based on TMA scoring. **C** Kaplan-Meier curves for overall survival from patients included in TMA according to the variations of BOK expression. Kaplan-Meier curves were statistically compared by the log-rank test. Source data are provided as a Source Data file. **D** BOK and MCL1 expression levels were analyzed by immunoblotting in the indicated breast cancer cell lines (*n* = 2). α-tubulin was used as a loading control. Scatter plot of IC_50_ of MBoIN179 compared with the relative expression of MCL1 (**E**) and BOK (**F**) in each breast cancer cell line analyzed. Each point is labeled with the legend in (**D**). Spearman coefficient and p-value are represented in each graph. MBoIN179 induces apoptosis in the HCC1954 cell line. **G** Cytotoxic effect of MBoIN179 on HCC1954 cells measured by MTS assay as an indirect measure of cell viability. Data represents the percentage of viable cells treated with MBoIN179 for 48 h at the indicated concentrations (12.5, 25, 37.5, 50, 62.5, 75, 87.5 and 100 µM). Data are presented as mean ± SD of three independent experiments. **H** BiFC measurement of the MCL1/BOK TMD hetero-interaction in HCC1954 cells in the presence of MBoIN179 (27 µM). Graph represents the mean ± SD, *n* = 6. One-way ANOVA followed by Dunnett’s multiple comparisons test displayed ****P < 0.0001. In the right panel, the protein expression level of the constructs is provided. HA epitope immunoblotting corresponds to VC-BOK TMD expression and myc epitope blotting to VN-MCL1 TMD. α-tubulin is used as a loading control. Quantification is provided in Fig. S2B. **I** MBolN179 localizes to the mitochondria. Mitochondrial fractions obtained by subcellular fractionation were analyzed by mass spectrometry, and spectra were background-corrected by subtracting signals from DMSO-treated controls. The main peak matches the expected molecular mass of MBolN179 (MW 352.5 Da). **J** Flow cytometry analysis of HCC1954 cells stained with Annexin V after treatment with MBolN179 (27 µM) overnight in the presence or absence of Z-VAD-fmk (5 µM). The percentage of Annexin V–positive cells was determined from 10,000 events analyzed per condition. **K** Flow cytometry analysis of mitochondrial membrane potential (TMRE) in HCC1954 cells following treatment with MBolN179 (27 µM) for 48 h. A total of 10,000 events were acquired per condition. FCCP reagent was used as a positive control for depolarization. Percentages indicate the proportion of cells with altered mitochondrial membrane potential. In both cases, the graph represents the mean ± SD of at least *n* = 4. One-way ANOVA followed by Dunnett’s multiple comparisons test revealed significant differences compared to the vehicle-treated group; **p < 0.01 and ****p < 0.0001. **L** Caspase 3/7 activity induced by MBoIN179 in HCC1954 control and BOK-silenced cells after 48 h of MBoIN179 (27 µM) treatment. Graph represents the mean ± SD, of *n* = 3. Student’s t-test was performed with a **p < 0.01 result. **M** Immunoblot and respective quantification of BOK-depleted 4T1, corroborating siRNA silencing in graph (**L**). Random siRNA (siRDM) was used as a negative control, and α-tubulin was used as a loading control. Graph represents the mean ± SD, of *n* = 3. Student’s t-test was performed with a **p < 0.01 result.

To assess the clinical relevance of BOK, we analyzed its expression by immunohistochemistry in a breast cancer cohort comprising 207 samples. Notably, BOK positivity was significantly associated with aggressive breast cancer subtypes, including HER2-enriched (64%) and triple-negative breast cancer (TNBC, 59%), compared with luminal subtypes (Luminal A: 7%, Luminal B: 17%; *p* < 0.001) (Fig. 3A, B). Moreover, its expression was also associated with lymph node (38% vs. 16%; *p* = 0.006) and distant metastasis (37% vs. 23%; *p* = 0.014). Consistently, BOK expression was higher in ER-negative (56%) and PR-negative (43%) tumours (both *p* < 0.001), while HER2 status alone did not reach statistical significance (Table 1). Importantly, Kaplan–Meier survival analysis revealed significantly worse overall survival in patients with BOK-positive tumours (Fig. 3C), establishing for the first time that BOK could be considered a novel prognostic marker of poor outcome in breast cancer. Paradoxically, these findings highlight a therapeutic opportunity: BOK expression in aggressive breast tumours could be exploited pharmacologically by releasing it from MCL1 inhibition, thereby converting a poor prognostic marker into a therapeutic vulnerability.

**Table 1:** Associations between BOK expression pattern and the clinicopathological variables in the cohort of 207 samples.

	BOK expression (%)		
	Negative	Positive	*p* Value
**Tumor grade**			
1 (*n* = 9)	5 (56%)	4 (44%)	.014
2 (*n* = 41)	37 (90%)	4 (10%)	
3 (*n* = 66)	45 (68%)	21 (32%)	
**Molecular subtype**			
Luminal A (*n* = 62)	58 (93%)	4 (7%)	<.001
Luminal B (*n* = 24)	20 (83%)	4 (17%)	
HER2 (*n* = 14)	5 (36%)	9 (64%)	
TNBC (*n* = 27)	11 (41%)	16 (59%)	
**Lymph node metastasis**			
No (*n* = 68)	57 (84%)	11 (16%)	.006
Yes (*n* = 55)	34 (62%)	21 (38%)	
**Distant metastasis**			
No (*n* = 101)	78 (77%)	23 (23%)	.014
Yes (*n* = 27)	17 (63%)	10 (37%)	
**Estrogen receptor**			
Negative (*n* = 43)	19 (44%)	24 (56%)	<.001
Positive (*n* = 84)	75 (89%)	9 (11%)	
**Progesterone receptor**			
Negative (*n* = 53)	30 (57%)	23 (43%)	<.001
Positive (*n* = 74)	64 (86%)	10 (14%)	
**HER2**			
Negative (*n* = 92)	71 (77%)	21 (23%)	n.s.
Positive (*n* = 34)	23 (68%)	11 (32%)	

To further investigate the involvement of MCL1 and BOK in breast cancer, we examined their expression and IC₅₀ values in a panel of murine and human breast cancer cell lines. (Fig. 3D–F). Interestingly, we observed a strong inverse correlation between MCL1 expression and MBoIN179 IC₅₀ values (Spearman’s ρ = –0.786 and –0.81 for XX and YY, respectively) (Fig. 3E, F). Higher levels of BOK and MCL1 were associated with increased sensitivity to the drug, reflecting a requirement for target availability to trigger cell death.

### Interference with the MCL1/BOK TMD interaction induces BOK-dependent apoptosis in breast cancer cells

Assuming that the MBoIN179 induces BOK translocation through MAMs, leading to MOMP, an apoptotic response would be expected. To explore this possibility, we first confirmed that treatment with MBoIN179 at the IC₅₀ was able to disrupt MCL1/BOK TMD interaction in the HER2-positive HCC1954 cell line using the BiFC assay previously described (Fig. 3G, H). Consistent with this, mass spectrometry analysis of mitochondrial fractions showed that the compound reaches mitochondria (Fig. 3I). Finally, activation of apoptosis upon treatment was confirmed using a combination of assays. We measured the exposure of phosphatidylserine on the outer leaflet of the plasma membrane, detected as an increase in FITC–annexin V binding by flow cytometry (Figs. 3J and S2C). Importantly, co-treatment with the pan-caspase inhibitor zVAD partially reduced annexin V staining, supporting that the observed effect is caspase-dependent (Fig. 3J). This effect was accompanied by mitochondrial depolarisation (Δψm), a consequence of MOMP (Fig. 3K). Finally, MBoIN179 induced BOK-dependent activation of the effector caspase-3 as demonstrated by the decrease in activity observed upon siRNA-mediated knockdown of BOK (Fig. 3L, M) that is recovered upon BOK overexpression (Fig. S2F). Moreover, MCL1 overexpression inhibits MBoIN179-induced apoptosis while MCL1ΔTM does not (Fig. S2G). These findings further support the on-target effect of MBoIN179 on MCL1/BOK TMD interaction.

**Fig. 4:**
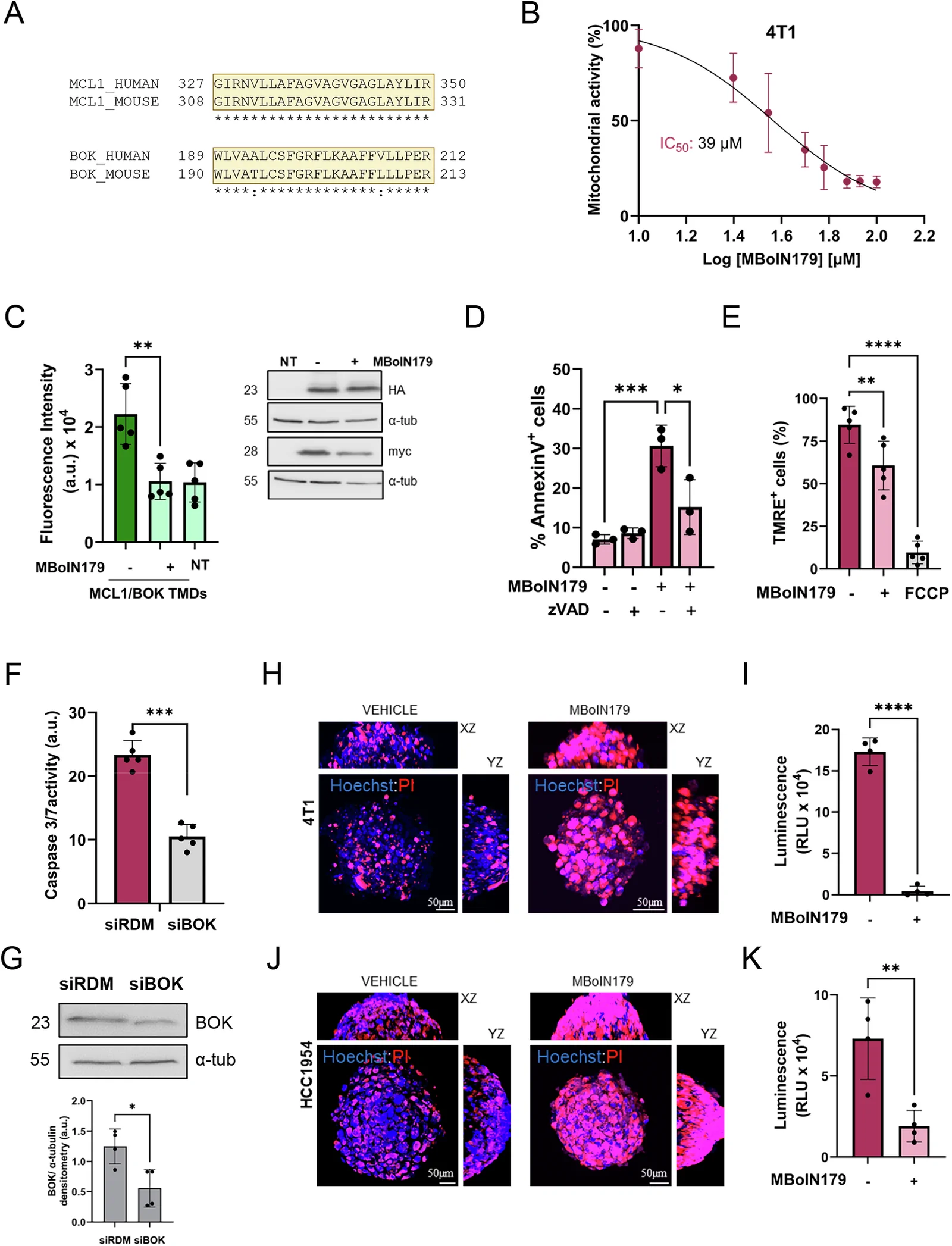
MBoIN179 induces apoptosis in the 4T1 breast cancer cell line. **A** Sequence alignment of human and mouse MCL1 and BOK TMDs. **B** Cytotoxic effect of MBoIN179 on 4T1 cells. Data represents the readout of the MTS assay as an indirect measure of cell viability. Cells were treated with MBoIN179 for 72 h at the indicated concentrations (10, 25, 35, 50, 60, 75, 85 and 100 µM). Percentages are relative to vehicle-treated cells. **C** Fluorescence quantification of BiFC signal of the MCL1/BOK TMD hetero-interaction in 4T1 cells in the presence of MBoIN 179 (40 µM). Graph represents the mean ± SD of at least *n* = 5. One-way ANOVA followed by Dunnett’s multiple comparisons test displayed significant differences compared to the vehicle-treated group; **p < 0.01. In the right panel, the protein expression level of the constructs is provided. HA epitope immunoblotting corresponds to VC-BOK TMD expression and myc epitope blotting to VN-MCL1 TMD. α-tubulin is used as a loading control. Quantification is provided in Fig. S2D. **D** Flow cytometry analysis of 4T1 cells stained with Annexin V after treatment with MBolN179 (40 µM) overnight in the presence or absence of Z-VAD-fmk (5 µM). The percentage of Annexin V–positive cells was determined from 10,000 events analyzed per condition. **E** Flow cytometry analysis of mitochondrial membrane potential (TMRE) in 4T1 cells following treatment with MBolN179 (40 µM) for 24 h. A total of 10,000 events were acquired per condition. FCCP reagent was used as a positive control for depolarization. Percentages indicate the proportion of cells with altered mitochondrial membrane potential. In both cases, graphs represent the mean ± SD, *n* = 4. One-way ANOVA followed by Dunnett’s multiple comparisons test displayed *p < 0.05, **p < 0.01 and ****p < 0.0001. **F** Caspase 3/7 activity induced by MBoIN179 in 4T1 control and BOK-silenced cells after 24 h of MBoIN179 (40 µM) treatment. Graph represents the mean ± SD, of *n* = 5. Student’s t-test was performed with a ***p < 0.001 result. **G** Immunoblot and corresponding quantification of BOK-depleted 4T1, corroborating siRNA silencing in graph (**F**). Random siRNA (siRDM) was used as a negative control and α-tubulin was used as a loading control. MBoIN179 induces cell death in 3D cancer models. **H** Representative image of 4T1-derived spheroids treated with vehicle or MBoIN179 (40 µM) for 72 h and stained with Hoechst (nuclei) and propidium iodide (PI; dead cells). Scale refers to 50 µm. **I** Luminescent signal (RLU, relative light units) corresponding to ATP quantification as a measure of cell viability in spheroids in (**H**) according to the CellTiter-Glo 3D assay. Graph represents the mean ± SD, of *n* = 3. Student’s t-test was performed with a ***p < 0.001 result. **J** Representative image of HCC1954-derived spheroids treated with vehicle or MBoIN179 (27 µM) for 72 h and stained with Hoescht (nuclei) and propidium iodide (PI; dead cells). Scale refers to 50 µm. **K** Luminescent signal (RLU, relative light units) corresponding to ATP quantification as a measure of cell viability in spheroids in (**J**) according to the CellTiter-Glo 3D assay. Graph represents the mean ± SD, of *n* = 3. Student’s t-test was performed with a **p < 0.01 result.

To enable in vivo evaluation, we first verified that the target TMDs are conserved between mouse and human by sequence alignment (Fig. 4A). We selected the 4T1 syngeneic mouse tumour model to enable evaluation of therapeutic responses in an immunocompetent setting. First, we corroborated the reduction of fluorescence in the MCL1/BOK BiFC TMD interaction assay at the IC_50_ (39 µM) (Fig. 4B, C) and the increase in the levels of Annexin V positive cells upon treatment (Figs. 4D and S2E) correlated with a decrease in the mitochondrial membrane potential (Fig. 4E). Treatment also induced BOK-dependent caspase-3 activation as confirmed by siRNA-mediated knockdown (Fig. 4F, G).

We studied the activity of MBoIN179 in 3D spheroid models, which more closely mimic the spatial complexity of tumours. To assess the ability of MBoIN179 to induce cell death in 3D spheroids, we measured the incorporation of propidium iodide (PI) (Fig. 4H) and ATP levels by CellTiter-3D-Glo Assay. This assay provides a luminescent signal proportional to the amount of ATP present, which is directly proportional to the number of viable cells. Treatment with MBoIN179 led to a marked reduction in ATP levels, consistent with loss of cell viability (Fig. 4I). Similar results were obtained in 3D spheroid models of HCC1954 cells (Fig. 4J, K).

**Fig. 5:**
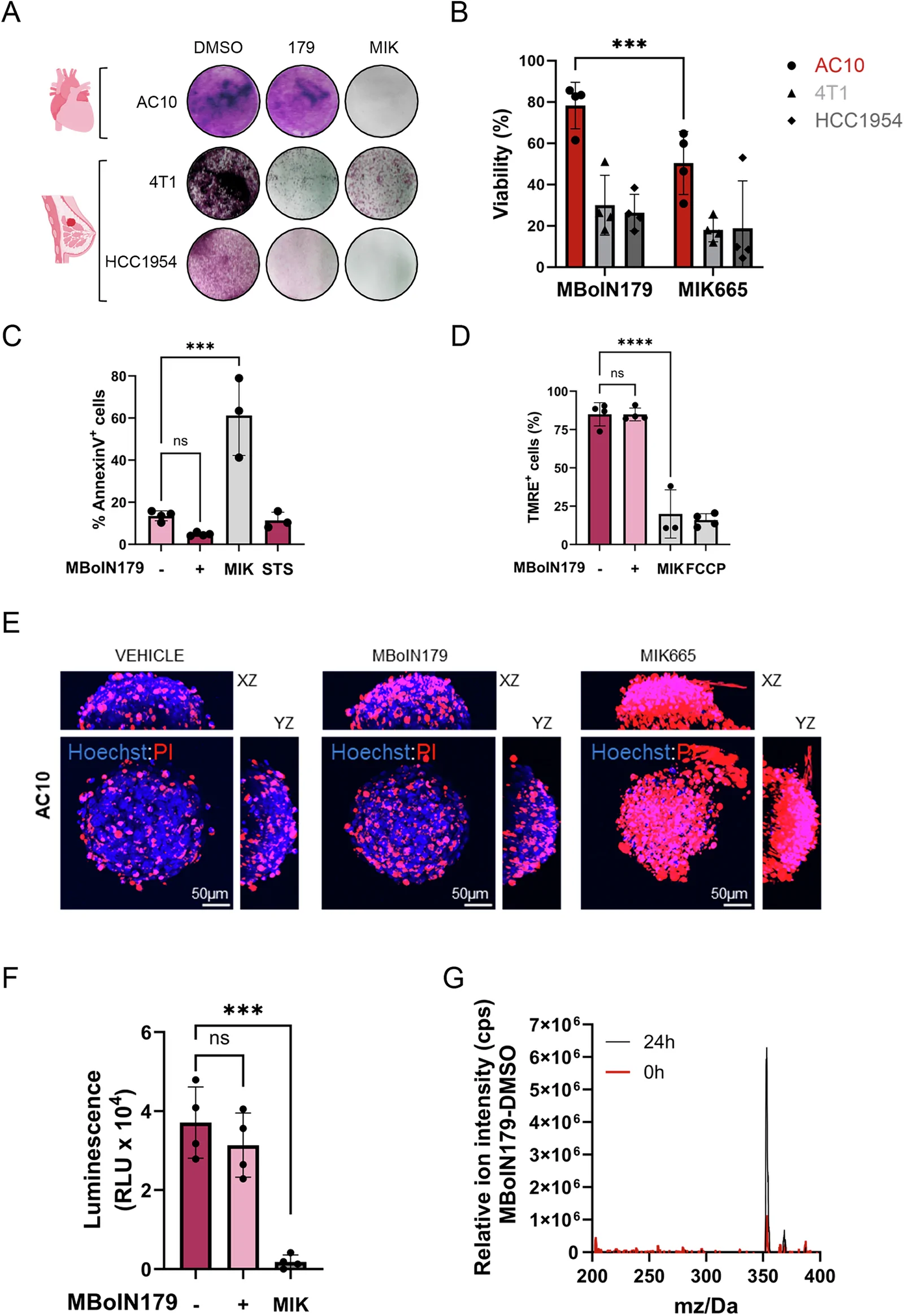
MBoIN179 demonstrates reduced cardiotoxicity in human cardiomyocytes. **A** Representative images of 4T1, HCC1954, and AC10 cells treated with vehicle (DMSO), MBolN179 (27 µM), or MIK665 (15 µM) for 48 h and stained with crystal violet after fixation. **B** Quantification of previous crystal violet plates. Data represented as mean ± SD, *n* = 4 and significant differences were analyzed using two-way ANOVA with Sidak’s post-hoc test (***p < 0.001). **C** Percentage of AC10 cells stained with Annexin V upon treatment with MBoIN179 (27 µM) and MIK665 (15 µM) for 48 h. **D** Mitochondrial membrane potential measurement induced by MBoIN179 (27 µM) and MIK665 (15 µM). FCCP reagent was used as a positive control for depolarization. In both cases, the graph represents the mean ± SD, *n* = 4. Data were analyzed using a one-way ANOVA followed by Dunnett’s post-hoc test; asterisks indicate statistical significance compared to the vehicle control group; ***p < 0.001 and ****p < 0.0001. **E** Representative image of AC10-derived spheroids treated with vehicle or MBoIN179 (27 µM) and MIK665 (15 µM) for 72 h and stained with Hoechst (nuclei) and propidium iodide (PI; dead cells). Scale refers to 50 µm. **F** Luminescent signal referred to ATP quantification as a viability measure for spheroids in (**E**) according to the CellTiter-3D-Glo assay. Graph represents the mean ± SD of *n* = 3. Student’s t-test was performed with a **p < 0.01 result. **G** MBolN179 accumulates in AC10 cardiomyocyte spheroids despite the absence of detectable toxicity. AC10 spheroids were treated with MBolN179 for 0 or 24 h, washed to remove extracellular compounds, and subjected to organic extraction followed by mass spectrometry analysis. Spectra were background-corrected by subtracting signals from DMSO-treated controls. The main peak corresponds to the expected molecular mass of MBolN179. An increase in peak intensity from 0 to 24 h indicates time-dependent incorporation of the compound into the spheroids.

To rule out the cardiotoxic effects associated with previously described MCL1 inhibitors, such as MIK665, we tested MBoIN179 in the human AC10 cardiomyocyte cell line. While MIK665 clearly induces cell death in human cardiomyocytes, MBoIN179 selectively triggers cell death in breast tumour cells without significantly compromising cardiomyocyte viability (Fig. 5A, B). In fact, increased levels of Annexin V staining (Fig. 5C) and mitochondrial depolarization (Fig. 5D) were also detected in AC10 in the presence of MIK665 but not when treated with MBoIN179, reinforcing our rationale. To confirm these results, we studied the behaviour of MIK665 and MBoiN179 in 3D cardiac spheroids. AC10-derived spheroids were treated for 72 h with MIK665 or MBoIN179, and then ATP levels were measured following CellTiter-3D-Glo Assay. Again, treatment with MIK665 but not MBoIN179 led to a marked reduction in ATP levels, consistent with loss of cell viability (Fig. 5E, F). Time-dependent accumulation of MBolN179 in AC10 spheroids, as detected by mass spectrometry, confirms efficient cellular uptake despite the lack of cardiotoxicity (Fig. 5G). In conclusion, our data indicate that targeting MCL1/BOK TMD interaction offers a benefit over classic MCL1 inhibition, as its wider therapeutic window enables the effective targeting of cancer cells without inducing cardiotoxicity.

### MBoIN179 promotes apoptosis and diminishes metastatic burden in an in vivo model of breast cancer

We next evaluated the efficacy of MBoIN179 in the 4T1 orthotopic, aggressive breast cancer model in BALB/c mice exhibiting high spontaneous metastatic potential. The 4T1 model spontaneously metastasizes from the primary tumour in the mammary gland to multiple distant sites, especially the lungs [35].

**Fig. 6:**
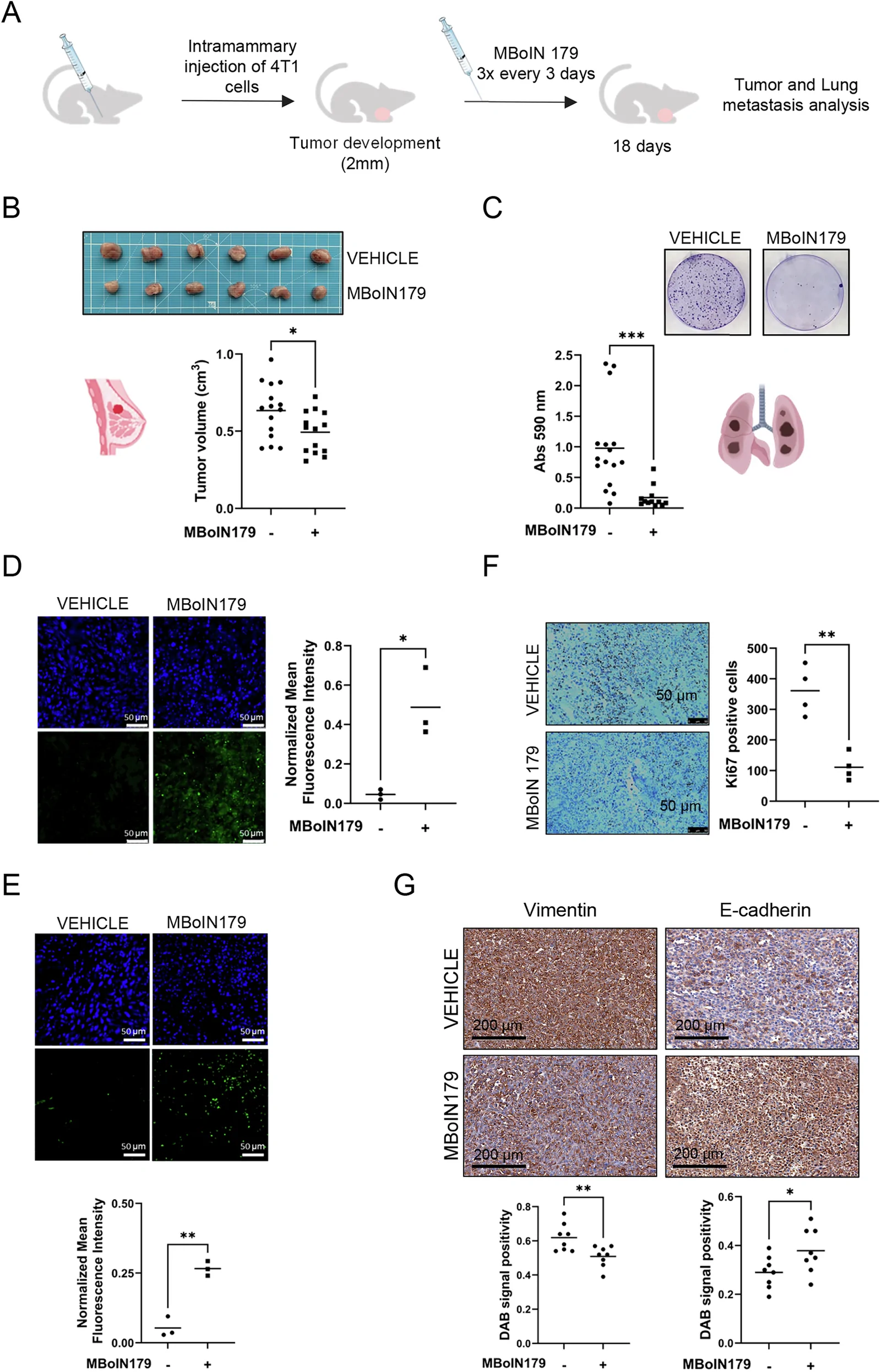
In vivo antitumour efficacy of MBoIN179 in the orthotopic allograft 4T1 model of breast cancer. **A** Scheme of the experimental procedure. **B** Changes in tumour volume from 4T1-engrafted mice treated with vehicle control or MBoIN179 at 75 mg/Kg three times for 18 days. Image represents tumours at the end of the experimental procedure. Data are presented as mean ± SD. Student’s t-test displayed *p < 0.05 versus vehicle. **C** Lung cells were plated in the presence of 6-TG for analysis of metastasis. A representative picture of the clonogenic assay with MBoIN179 treatment and quantification of all the plates is shown. Graph data are presented as mean ± SD. Student’s t-test displayed ***p < 0.001 versus vehicle. **D** Activation of apoptosis in treated tumours. Caspase-3 (green) immunostaining of 4T1 tumour tissues derived from mice treated or not with MBoIN179 as indicated in (**A**). Nuclei were stained with DAPI (blue). Green fluorescence was quantified using the ImageJ software (right panel). Scale bar 50 μm. Student’s t-test displayed *p < 0.05 versus vehicle. **E** BOK (green) immunostaining of 4T1 tumour tissues derived from mice treated or not with MBoIN179 as indicated. Nuclei were stained with DAPI (blue). Green fluorescence was quantified using the ImageJ software (upper panel). Scale bar 50 μm. Student’s t-test displayed **p < 0.01 versus vehicle. **F** Immunohistochemical detection of Ki67 in sections of tumours at the experimental endpoint with and without MBoIN179 treatment. Scale bar 50 μm. Data are presented as mean ± SD of five fields of 3 different tumours. Student’s t-test displayed **p < 0.01 versus vehicle. **G** Immunohistochemical detection of Vimentin and E-cadherin in sections of tumours at the experimental endpoint with and without MBoIN179 treatment. Scale bar 200 μm. Data are presented as mean ± SD of five fields per tumor (8 tumors per group). Statistical significance was determined by Student’s t-test (*p < 0.05, **p < 0.01 vs. vehicle).

First, we determined the maximum tolerable dose of MBoIN179 and evaluated its potential toxicity. Notably, the compound exhibited no overt toxicity in vivo (Fig. S3) at the maximum soluble dose of 75 mg/kg. Comprehensive haematological analyses revealed no significant alterations in white blood cell count, red blood cell indices, haemoglobin levels, or platelet numbers (Fig. S3A, B). In the same line, biochemical profile (Fig. S3C) was not affected by MBoIN179, supporting a favourable systemic safety profile. Based on these findings, tumour-bearing mice were treated intraperitoneally with MBoIN179 at 75 mg/kg. (Fig. 6A). Treated animals showed no treatment related mortality (Fig. S3D), maintained stable body weight (Fig. S3E) and mice preserved cardiac histoarchitecture (Fig. S3F).

Notably, mice treated with MBoIN179 showed a significant reduction in both tumour growth and metastatic dissemination (Fig. 6B, C). Immunofluorescent staining for active caspase-3 in tumour sections revealed a marked increase in apoptotic cell death in the MBoIN179-treated animals (Fig. 6D), which correlates with an increase of BOK expression (Fig. 6E). Furthermore, the immunohistochemical analysis of Ki67 demonstrated a substantial decrease in proliferative activity in tumours from MBoIN179-treated mice (Fig. 6F). It is worth noting that, tumours exhibited a decrease in vimentin mesenchymal marker and an increase in E-cadherin epithelial marker, suggesting reversion of epithelial-mesenchymal transition (EMT) and reduction of the invasive capacity, which correlates with the observed decrease in lung-metastasis (Fig. 6G). Altogether, these findings demonstrate that MBoIN179 inhibits the growth of breast tumours and metastasis without causing harmful side effects in TNBC preclinical models.

This in vivo validation underscores the value of targeting BOK/MCL1 TMD as a novel therapeutic approach in breast cancer.

## Discussion

In this study, we report the identification of a novel chemical disruptor that interferes with the BOK/MCL1 mitochondrial transmembrane protein-protein interaction, resulting in robust induction of breast cancer cell death in vitro and in vivo, while preserving cardiomyocyte viability.

Regarding cardiotoxicity, it is worth noting that, unlike the canonical effectors BAX and BAK, BOK is expressed at markedly lower levels in cardiac tissue [36, 37], suggesting that BOK is less relevant for apoptotic control in cardiac tissue compared to the effectors BAX and BAK.

Using a reconstituted LUV-based system that captures the regulatory interplay between MCL1 and BOK, we provide direct evidence that MBolN179 counteracts MCL1-mediated inhibition of BOK pore formation. To elucidate how MBoIN179 restores BOK pore activity at the lipid membrane, we examined its thermodynamics and residue-level interactions using atomistic MD simulations. Free energy calculations revealed a strong driving force for membrane partitioning: the free energy profile exhibits a deep minimum of ~10 k_B_T at 0.9 nm from the bilayer center, separated from bulk water by only a shallow interfacial barrier. Thus, under physiological conditions, MBoIN179 is expected to accumulate to substantially higher effective concentrations within the hydrocarbon core, positioning it to interact with membrane-embedded protein surfaces. Consistent with this, unbiased MD simulations showed that MBoIN179 can form hydrophobic contacts with the transmembrane helices of both MCL1 and BOK. Strikingly, many of these interactions overlap with residues previously mapped to the BOK–MCL1 heterodimer interface [17]. This correspondence indicates that MBoIN179 preferentially samples the same membrane-embedded surface used for TMD–TMD association, where it may disrupt helix–helix packing—either by steric occlusion or by modulating local lipid–protein interactions—thereby destabilizing the heterodimer. Together, the free-energy landscape and contact-mapping analyses provide a coherent mechanistic explanation for the experimental observations: MBoIN179 is thermodynamically driven to enrich within the bilayer interior and selectively engages residues that mediate the MCL1/BOK TMD interaction, consistent with the BiFC loss-of-signal and the restored BOK pore activity in reconstituted systems. These membrane-centric mechanisms support the feasibility of pharmacologically targeting pathogenic TMD interfaces with small molecules.

Developing small molecules that target transmembrane protein-protein interactions remains highly challenging and scarcely explored [38]. However, emerging evidence demonstrates that these interfaces are not undruggable. Small molecules such as Vorapaxar, a PAR-1 antagonist that binds deep within the transmembrane bundle to disrupt receptor activation [39], and Ivacaftor, a CFTR potentiator that stabilizes specific transmembrane conformations of the chloride channel [40], exemplify how chemical ligands can allosterically modulate membrane-embedded domains with therapeutic benefit. Recently, Np75-4A22, a small molecule capable of interacting with the TMD of the p75^NTR^ receptor, preventing the spread and migration of melanoma cells, has been described [41]. Even though transmembrane interactions are notoriously difficult to study, these evidences, together with our results, confirm the specific druggability of TMD.

Predominantly localized to the ER, BOK requires translocation to mitochondria for classical pro-apoptotic activity [22]. Our results show that the disruption of MCL1/BOK-TMD interaction promotes the increase of MAMs and the trans-organellar movement of BOK to the mitochondria, where it induces apoptosis. The role of BOK and MCL1 in MAMs dynamics has been previously reported [19, 42, 43]. In fact, murine embryonic fibroblasts deficient for BOK have fewer MAMs and altered protein composition [42]. The requirement for trans-organelle trafficking of BOK across MAMs may function as a critical checkpoint in apoptosis. Such spatial restriction likely prevents the premature accumulation of BOK at the mitochondrial outer membrane, where its pore-forming potential could otherwise trigger uncontrolled cell death. The inhibitory interaction with MCL1 may further stabilize this regulatory node, reinforcing MAMs as signaling hubs that integrate stress cues and safeguard mitochondrial integrity. In this context, BOK might operate as a sensor of ER homeostasis, linking ER stress dynamics to mitochondrial apoptotic priming. Notably, MBoIN179 appears to bypass this checkpoint, promoting the translocation of BOK to the mitochondrial membrane, thereby inducing apoptosis in tumour cells. Although BOK silencing significantly reduces caspase activation, it does not completely abolish it, which is consistent with the partial knockdown achieved in our system (~60–70%). Residual BOK expression is likely sufficient to sustain a degree of apoptotic signaling, particularly considering the strong amplification and processivity of the caspase cascade. In addition, while BOK is reduced, MCL1 remains present. Therefore, we cannot discard that modulation of other MCL1 transmembrane interactions may contribute to the residual activity observed upon BOK silencing.

Interestingly, the analysis of BOK protein expression in breast cancer patient samples reveals that high BOK protein expression is significantly associated with aggressive phenotypes (HER2-enriched and TNBC), lymph node and distant metastasis, and hormone receptor negativity. Importantly, Kaplan–Meier survival analysis demonstrated that patients with BOK-positive tumours exhibit markedly worse overall survival compared to BOK-negative cases, establishing BOK as a novel prognostic marker of poor outcome in breast cancer. This paradox underscores the context-dependent nature of BOK biology and highlights a therapeutic opportunity: although BOK marks aggressive disease, its presence can be exploited pharmacologically by disrupting the MCL1–BOK interaction, thereby converting a marker of poor prognosis into a vulnerability for apoptosis induction.

Our in vivo breast cancer studies show that MBoIN179 treatment activates caspase-3 and reduces expression of the proliferation marker Ki-67, resulting in a significant decrease in tumour size. Notably, a decrease in metastasis associated with increased E-cadherin expression and a decrease in vimentin levels is observed. These data suggest that MBoIN179 not only promotes apoptotic cell death but also restrains epithelial–mesenchymal transition (EMT). This dual activity may be particularly relevant in the context of metastatic progression. For BOK, the molecular target of our drug, a role in EMT regulation has already been reported [44], supporting the idea that its pharmacological inhibition can simultaneously trigger cell death and prevent phenotypic plasticity. Such convergence of apoptotic induction and EMT blockade could therefore represent a key therapeutic advantage, limiting both tumour survival and dissemination.

Altogether, the data suggest that the MBoIN179’s in vivo mechanism of action operates via BOK release from MCL1. The dual action of MBoIN179-BOK release to induce apoptosis coupled with EMT blockade may yield deeper and more durable responses across aggressive tumour contexts. Importantly, the selective expression of BOK in tumour tissues, but not in cardiac tissue, may provide a therapeutic advantage by allowing efficient tumour elimination while minimizing potential cardiotoxic effects. This work delineates a clear future preclinical trajectory to convert current hits into bona fide leads and to evaluate the therapeutic scope of BOK as a target across different tumour types.

## Material and methods

### Human breast cancer and immunohistochemical analyses

A total of 207 breast carcinoma (BC) tumours were included in this study. Samples were retrospectively obtained from the MD Anderson Foundation Biobank Spain (record number B.0000745, ISCIII National Biobank). Patients underwent surgical resection between 2010 and 2017, with a mean age at surgery of 52.9 years (range, 44–83 years). Histological and immunohistochemical analyses were performed on formalin-fixed, paraffin-embedded tissue samples. Two 1-mm cores from each tumour block were used to construct tissue microarrays (TMAs). Clinical and pathological characteristics of the cohort are summarized in Table S1. Breast cancer intrinsic subtypes were defined as previously described [45]. The study was conducted in accordance with Spanish biomedical research regulations (Ley de Investigación Orgánica Biomédica, 14 July 2007; Real Decreto Biobancos 1716/2011) and was approved by the ethics committee of Hospital Ramón y Cajal (Madrid, Spain).

#### Immunohistochemical analyses

Immunohistochemical staining for estrogen receptor (ER), progesterone receptor (PR), and human epidermal growth factor receptor 2 (HER2), as well as BOK expression, was performed using an automated Leica BOND platform (Leica Biosystems), according to the manufacturer’s instructions. Sections (2 μm thick) were deparaffinized and rehydrated on the instrument, followed by heat-induced epitope retrieval using Leica BOND Epitope Retrieval Solution (ER2) for ER (Clone 6F11, Leica), PR (PA0312, Leica), HER2 (BMA-L-RUO, Leica) and BOK (ab233072, Abcam) for 20 min. Breast tumours were classified according to immunohistochemical assessment of ER, PR, and HER2, according to current ASCO/CAP guidelines. ER and PR positivity were defined as nuclear staining in ≥1% of tumour cells. HER2 status was determined by immunohistochemistry, with scores of 3+ considered positive, and equivocal (2 + ) cases further evaluated by in situ hybridization. Tumours were classified as BOK-positive (staining in ≥10% of tumour cells) or BOK-negative.

Associations between BOK expression status (BOK-positive vs BOK-negative) and clinicopathological variables were evaluated with SPSS 30.0.0.0 (172) using the χ² test or Fisher’s exact test, as appropriate. Overall survival was defined as the interval between the date of surgery and death from any cause or last follow-up. Survival curves were generated using the Kaplan–Meier method and compared using the log-rank test. *P* values < 0.05 were considered statistically significant.

### Bimolecular fluorescence complementation-TMD assays

Bimolecular fluorescence complementation (BiFC) analysis was performed as previously described [17]. The GpA, MCL1, BID, and BOK TMDs were cloned at the C-terminal end of Venus protein fragments (N-term - VN and C-term - VC), maintaining their natural topology in full-length proteins. HCT116 cells were seeded in six-well plates (300000 cells/well) and co-transfected with 0.5 μg of VN and 0.5 μg VC DNA constructs using TurboFect (Thermo Scientific™) according to manufacturer instructions. For Venus fluorescence measurement, cells were harvested and resuspended in 150 µl phosphate-buffered saline (PBS) after 16 h transfection. Fluorescence emission was measured using 96-well black plates and a Wallac 1420 Workstation (ʎexc 510 nm and ʎem 535 nm). MBoIN179 was obtained from Ambinter and dissolved in DMSO. Concentrations used are detailed in the figure legends. For representative confocal cell images, cells were transfected, as previously described, on a microscope cover glass (24 mm diameter) stained with Hoechst for nuclei detection and maintained in DMEM at 37 °C and 5% CO2 for imaging. Image acquisition used a Leica SP8 confocal microscope. Excitation light came from 405 nm and 448 nm lasers.

### Primary high-throughput screening (1HTS)

An image-based high-throughput screening assay was developed to identify inhibitors of MCL1/BOK TMDs hetero-oligomerization, based on the BiFC TMD assay. The screening was performed in a 96-well format, where HCT116 cells (25,000) were seeded and after 24 h, transfected using Turbofect with 0.1 µg of each plasmid (VN and VC) according to the manufacturer’s instructions. Four hours after transfection, compounds from the MyriaScreen library (Sigma-Aldrich) or DMSO (to the positive and negative controls) were added to the assay plate to a final concentration of 50 µM in 2% DMSO. Fluorescence images were acquired after 16 h, using a Leica TCS SP8 confocal microscope. The Z’ factor for most assay plates was greater than 0.5, indicating a robust assay performance. Venus and Hoechst mean fluorescence intensities (MFIs) were quantified, and the ratio Venus MFI/Hoechst MFI was calculated to get the normalized MFI per well.

### Secondary high-throughput screening (2HTS)

The 2HTS assay was set up to identify false positives. Using the same conditions as for 1HTS, cells were transfected with Glycophorin A (GpA) TMD, cloned in the BiFC system as a control for unrelated transmembrane interactions, and VENUS protein as a BiFC control. Treatments with the positive compounds from the 1HTS were carried out at a final concentration of 50 µM in 2% DMSO, as described above. Fluorescence images and normalised MFI per well were obtained using the same procedure as previously described.

### Cell culture

All cell lines used in this study were recently obtained from ATCC, tested for mycoplasma contamination, and maintained in an incubator at 37 °C, 5% CO2 and 95% of humidity. Human colorectal carcinoma cells (HCT116) were grown in McCoy’s 5 A medium (Gibco) supplemented with 10% fetal bovine serum (FBS; Sigma-Aldrich) and 1% penicillin/streptomycin (Gibco). Human ductal carcinoma cells (HCC1954) were grown in RPMI medium 1640 (Gibco) supplemented with 10% FBS and 1% penicillin/streptomycin. Human adenocarcinoma breast cancer line MCF-7 and human cardiomyocytes AC10 were grown in DMEM F12 (Gibco) supplemented with 10% FBS and 1% penicillin/streptomycin. MDA-MB-468, MDA-MB-231 and Mus musculus breast cancer cell lines 4T1 and EO771 were grown in DMEM (Gibco) with 10% FBS and 1% penicillin/streptomycin.

### In situ proximity ligation assays (PLA)

Mitochondria-associated membranes (MAMs) were detected using in situ proximity ligation assays, a method enabling the visualization of endogenous protein-protein interactions [32]. To conduct the Duolink PLA (Sigma Aldrich) assay, HCT116 cells were seeded on microscope coverglass (12 mm diameter), at a density of 50,000 cells per well and treated with MBoIN179 at 50 µM. The experimental procedure follows the manufacturer’s protocol. The primary antibodies used were: VDAC1 (ab14734, Abcam, mouse) and IP3R (ab5804, Abcam, rabbit) IP3R (ab252536, Abcam, mouse) and BOK ([R1-5-1] ab233072, Abcam). Coverslips were stained with Duolink in situ mounting medium with DAPI. Cell images were acquired using a Leica TCS SP8 confocal microscope. Excitation light came from 405 nm and 552 nm lasers. Image processing was performed using Fiji software. For each condition (DMSO and MBolN179), 10 random fields per experiment were analyzed in three independent experiments. In each field, nuclei (blue signal) and MAMs (red puncta) were quantified, and the ratio of MAMs to nuclei was calculated. Data are presented as the mean number of MAMs per cell.

### Subcellular fractionation

HCC1954 cells (7 × 10^6^) treated with MBoIN179 at the concentration and times indicated in the figure were harvested, washed in cold PBS, and resuspended in cold SEM buffer (10 mM HEPES, pH 7.2, 250 mM sucrose). Cells were mechanically lysed by passing them through a 25 G needle twelve times. Nuclei and cellular debris were removed by centrifugation at 500 × *g* for 5 min at 4 °C. The post-nuclear supernatant was centrifuged at 10,000 × *g* for 15 min at 4 °C, and the heavy membranes (pellet) fraction was separated.

### Recombinant protein expression and purification

The full-length human BOK gene (FL-BOK) was cloned into the pETM-11 expression vector (EMBL), containing an N-terminal 6×His tag. For expression of MCL1, a truncated form lacking the first 178 amino acids (residues 178–350) was cloned into pET30a, generating an N-terminal 6×His–maltose-binding protein (MBP) fusion (6×His-MBP-MCL1^S178-R350^, from now on referred to as MCL1^S178-R350^). Both constructs were transformed into Escherichia coli expression strains: BL21(DE3)-RIPL for FL-BOK and BL21(DE3)-CodonPlus-RIL (Agilent Technologies) for MCL1 MCL1^S178-R350^. Bacterial cultures were grown in LB medium supplemented with the appropriate antibiotics and induced with 1 mM isopropyl-β-D-thiogalactoside (IPTG) at OD₆₀₀=1.0. Induction was carried out for 4 h at 18 °C. Cells were harvested by centrifugation and resuspended in lysis buffer. For FL-BOK, lysis buffer consisted of 20 mM Tris-HCl, 1 M NaCl, and 1 mM PMSF (pH 8.0); for MCL1^S178-R350^, 25 mM Tris-HCl, 300 mM NaCl, and 1 mM PMSF (pH 8.0). In both cases, cells were lysed by sonication and clarified by centrifugation at 14,000 × *g* for 1 h at 4 °C. The soluble fractions were loaded onto Ni-NTA agarose resin (Qiagen), and bound proteins were eluted with 250 mM imidazole in buffer. Eluted proteins were concentrated and further purified by size-exclusion chromatography using a Superdex 75 Increase 10/300 GL column (Cytiva) equilibrated in 20 mM HEPES, 150 mM KCl, pH 8.0. Protein purity was assessed by immunoblotting, and concentrations were determined by NanoDrop spectrophotometry (Thermo Fisher Scientific). Protein identity was confirmed by mass spectrometry.

### Mass spectrometry analysis

Mitochondrial fractions from HCC1954 cells were obtained as previously described and subjected to organic extraction by resuspension in acetonitrile containing 0.1% formic acid, followed by sonication for 30 min. Samples were then centrifuged at 15,000 × *g* for 10 min at 4 °C, and the supernatant was collected and dried under a nitrogen stream. The dried residue was resuspended in 50 µL of water: acetonitrile (95:5, 0.1% formic acid) prior to mass spectrometry analysis.

AC10 spheroids were incubated with MBolN179 (3 µM) for the indicated times (0 and 24 h). Following treatment, spheroids were collected and washed with PBS containing 1% BSA, followed by two additional washes in PBS to remove extracellular compounds. Control spheroids were treated with vehicle (DMSO) under identical conditions. Samples were lysed, and organic extraction was performed as described above.

For in vivo studies, hearts were collected from mice treated with MBolN179 (75 mg/kg) for 1 h. After sacrifice, hearts were washed with PBS, snap-frozen in liquid nitrogen, and homogenized under cryogenic conditions using a mortar and pestle. The resulting homogenate was resuspended in acetonitrile containing 0.1% formic acid, and organic extraction was performed as described above.

Samples were analyzed by mass spectrometry using an AB Sciex Exion LC- Qtrap 4500 instrument operated in positive ion mode over an m/z range of 200–400. Spectra from MBolN179-treated samples were background-corrected by subtraction of the corresponding DMSO control spectra. The presence of MBolN179 was assessed by monitoring the ion signal corresponding to its expected molecular ion. For spheroid experiments, signal intensity at 24 h was compared to the 0 h condition to evaluate time-dependent intracellular accumulation. Representative spectra are shown as normalized ion intensity versus mass-to-charge ratio (m/z).

### Liposome permeabilization assay

Lipids were purchased from Avanti Polar Lipids and dissolved in chloroform. For liposome preparation, phosphatidylcholine and cardiolipin were mixed at a molar ratio of 8:2. The lipid mixture was dried under vacuum for 3 h to form a thin lipid film. As previously described [22], large unilamellar vesicles (LUVs) were generated by hydrating the lipid film with 80 mM calcein in buffer (pH 7.0) to a final lipid concentration of 5 mg/mL, followed by ten freeze–thaw cycles. The suspension was extruded through a 100 nm polycarbonate membrane using a mini-extruder (Avanti) and glass syringes. Unencapsulated dye was removed via gel filtration using Sephadex G-50. For the permeabilization assay, calcein-loaded LUVs were incubated with serial dilutions of recombinant proteins in black 96-well plates (PerkinElmer), and fluorescence was measured at 520 nm (excitation at 490 nm) over 1 h using a Victor² plate reader (PerkinElmer). Total dye release was determined by treatment with 0.1% Triton X-100, and the percentage of calcein release was calculated using the formula:

$${Calcein}\,{release}\, \% =\frac{{F}_{{sample}}-{F}_{{buffer}}}{{F}_{{Triton}}-{F}_{{buffer}}}x\,100$$

### Fluorescence polarization assay

Fluorescence polarization assays were performed using a carboxyfluorescein-labeled BAK BH3 peptide (CF-Bak). CF-Bak (20 nM) was incubated with recombinant MCL1ΔTM (E322), ensuring peptide binding. Increasing concentrations of MIK665 or MBolN179 were then added to the pre-formed complex and incubated at room temperature for 10 min. Polarization was measured using a plate reader (Ex 485 nm/Em 535 nm). A decrease in polarization was interpreted as the displacement of CF-Bak from MCL1.

### Molecular dynamics simulations

Molecular dynamics simulations were performed using GROMACS 2022 [46]. Atomistic models employed the CHARMM36m force field (July 2021 release) for proteins and lipids, the CHARMM TIP3P water model, and CHARMM36 parameters for ions [47, 48]. Parameters for MBolN179 were generated using the CHARMM-GUI Ligand Reader & Modeler web server [49]. All simulations were carried out in the isothermal–isobaric (NpT) ensemble using a 2 fs integration time step. Temperature was maintained at 310 K with a Nosé–Hoover thermostat (τ = 1 ps) [50], applying separate coupling groups for solute (peptides, drug, and lipids) and solvent (water and ions). Pressure was controlled at 1 bar using a Parrinello–Rahman barostat (τ = 5 ps) [51] with semi-isotropic coupling and a compressibility of 4.5 × 10⁻⁵ bar⁻¹ in both the lateral and normal directions.

The Verlet cutoff scheme [52] was employed with a neighbor-list update frequency of 20 steps and a real-space cutoff rlist = 1.2 nm. Electrostatics were treated with the particle-mesh Ewald (PME) [53] method using a real-space cutoff of 1.2 nm. Lennard–Jones interactions were computed using a force-switch modifier between 1.0 and 1.2 nm (rvdw_switch = 1.0 nm; rvdw = 1.2 nm). Bond constraints involving hydrogens were applied using the LINCS algorithm [54].

We performed two complementary sets of all-atom MD simulations: (1) Unbiased binding simulations to characterize how MBolN179 interacts with the transmembrane (TM) segments of Bok and MCL1; (2) Biased free-energy simulations using umbrella sampling to quantify the membrane partitioning of MBolN179.

#### Unbiased simulations

The α-helical TM segments of BOK (^328^IRNVLLAFAGVAGVGAGLAYLIR^350^) and MCL1 (^183^SGRLRSHWLVAALCSFGRFLKAAFFVLLPER^213^), previously generated using the online ProBuilder tool (https://www.ddl.unimi.it/vegaol/probuilder.htm) and pre-equilibrated for 1 μs in a membrane environment in our earlier work [17], were embedded in a 150-lipid POPC bilayer generated using the CHARMM-GUI server [55]. Each membrane was solvated with water and supplemented with 0.15 M KCl to neutralize the system and reproduce physiological ionic strength. A single MBolN179 molecule was then placed either in the aqueous phase or pre-inserted into the membrane at three distinct positions to generate independent replicas. All systems were simulated for 500 ns.

#### Free-energy calculations

To characterize membrane partitioning, one MBolN179 molecule was pulled from bulk water to the bilayer midplane of a POPC membrane. Umbrella sampling was carried out using 51 windows spaced at 0.1 nm intervals, with each window simulated for 100 ns. In each window, the molecule was restrained along the bilayer normal (z-axis) using a harmonic potential with a force constant of 2000 kJ·mol⁻¹·nm⁻², while lateral (xy-plane) motion was left unrestricted. The potential of mean force (PMF) along z was reconstructed using the GROMACS implementation of Weighted Histogram Analysis Method [56] based on the biased distance distributions and the known umbrella force constant, using the final 50 ns of each trajectory. Statistical uncertainties were estimated by 200 bootstrap iterations. The PMF zero was set in bulk water.

### Gel electrophoresis and immunoblotting

Whole-cell extracts were subjected to SDS-PAGE, transferred to nitrocellulose membranes, and blotted following standard procedures. Briefly, membranes were blocked for 1 h with 5% skimmed milk and then probed with primary antibodies at 4 °C overnight in 5% skimmed milk diluted 1:1000. After 3 washes with TBS-T, membranes were incubated for 1 h at RT with peroxidase-labeled secondary antibodies specific for each IgG (diluted 1:3000, purchased from Sigma-Aldrich). Primary antibodies used: rabbit monoclonal anti-HA (clone Y-11, no. H6908), mouse monoclonal anti-c-myc (clone 9B11, no. 2276S), rabbit polyclonal anti-MCL1 (no. 4572), rabbit anti-BCLXL (clone 54H6, no. 2764) from Cell Signaling; rabbit monoclonal [EPR15331] anti-BOK (ab186745) and rat anti-tubulin YL1/2 (ab6160) from Abcam. Immunoblot signals were detected using GE Healthcare’s ECL detection reagents and acquired in an ECL™ Detection System.

### Caspase 3/7 activity

HCC1954 and 4T1 cells were plated in six-well plates at 250,000 cells/well for 24 h. The cells were then transfected with 1 µg total VN plasmid or 0.5 µg VN construct plus 0.5 µg pcDNA3.1 plasmid using TurboFect (Thermo Scientific™), according to manufacturer instructions. After 16 h protein expression, cells were harvested, and the pellets were resuspended in lysis buffer (50 mM PIPES pH 7, 50 mM KCl, 5 mM EGTA, 2 mM MgCl_2_ and protease inhibitor cocktail). The pellets were frozen and thawed three times using liquid nitrogen, and cell lysates were then centrifuged at 13,200 rpm for 10 min. The supernatants were collected, and total protein concentration was quantified by using the bicinchoninic acid (BCA) method. For caspase 3/7 activity, a total of 50 μg protein in caspase assay buffer (PBS, 10% glycerol, and 2 mM DTT) containing 20 μM Ac-DEVD-AFC was monitored using a Wallac 1420 Workstation (λ_exc_ 390 and λ_em_ 510 nm).

### Cell toxicity assay

Cell viability was measured by a 3-(4,5-dimethylthiazol-2-yl)-5-(3-carboxymethoxyphenyl)-2-(4-sulfophenyl)-2H-tetrazolium (MTS) assay, using the CellTiter 96® Aqueous Non-Radioactive Cell Proliferation Assay kit (Promega). 4T1 or HCC1954 cells were grown in 96-well plates at a cell density of 7,5 × 10^4^ cells/well. After 24 h, cells were treated with indicated concentrations of MBoIN179 and 48 h later, cell viability MTS assay was performed according to the manufacturer's instructions. Plates were read at 490 nm on a Wallac 1420 workstation.

### Annexin V/PI staining

Cells were labeled with Alexa fluorescein isothiocyanate-conjugated Annexin V (BD Bioscience, San Jose, CA, USA) together with propidium iodide (PI) dead cell counterstain, according to the manufacturer’s recommendations, in binding buffer (10 mM HEPES, pH 7.4, 140 mM NaCl, 2.5 mM CaCl2). Staining for Annexin V and PI was assessed by flow cytometry on a CytoFLEX flow cytometer, followed by data analysis using CytExpert software (Beckman Coulter Life Sciences).

### TMRE staining

Cells transfected in the above-described conditions were incubated with 50 nM TMRE dye (Invitrogen, T669) at 37 °C/5% CO2 for 30 min. Then, samples were analyzed in a CytoFLEX flow cytometer. As a positive control of mitochondrial depolarization, cells pretreated with 20 μM FCCP for 10 min were used.

### BOK silencing

4T1 or HCC1954 cells were plated in six-well plates at a concentration of 200,000 cells/well. Cells were transfected with the On-Target plus Human BOK siRNA (Dharmacon). Transfection solution (OPTIMEM without supplements) contained 2.5 µl Lipofectamine 2000 (Invitrogen) and 100 µM siRNA. After 24 h, cells were transfected with 1 µg of the VN plasmids, and cell extracts were processed after 20 h of expression.

### Spheroids

HCC1954, AC10 and 4T1 cells were seeded at a density of 1000 cells per well in Corning® 96-well Clear Round Bottom Ultra-Low Attachment Microplates. Cells were allowed to grow for 4 days to form spheroids, after which they were treated with MBoIN179 at the IC_50_. Treatments were maintained for 72 h, followed by assessment of cell viability using the CellTiter-Glo 3D Cell Viability Assay Kit (Promega). Additionally, spheroids were stained with propidium iodide and Hoechst (1 µg/mL) and imaged using a Leica SP8 MP Intravital multiphoton microscope.

### Mouse 4T1 breast tumour model

All animal work was conducted according to protocols approved by the Animal Ethical Committee (2021/VSC/PEA/0255 & 2022/VSC/PEA/0091) from Conselleria de Agricultura, Agua, Ganadería y Pesca, Generalitat Valenciana. Sample size was calculated using GRANMO Software. Animals were maintained in ventilated racks under pathogen-free conditions at Principe Felipe Research Centre (Valencia, Spain), with food and water ad libitum and alternate dark and light cycles.

Eight-week-old female BALB/c mice, purchased from Janvier Labs, were injected subcutaneously in the second inferior mammary gland with 750,000 4T1 cells suspended in 0.1 mL PBS. Tumour growth was controlled every two days using an electronic caliper, and volume (mm3) was estimated using the formula (length x width2)/2, where width was considered to be the shorter of two perpendicular diameters. Moreover, weight and animal welfare were also evaluated throughout the procedure, attending to Morton and Griffiths scale criteria. When the tumour reached 2 mm in size, the MBoIN179 treatment was performed. Specimens were distributed randomly in the animal facility using non-automated simple random allocation. The study was conducted under an open-label design without blinding. Then, mice were injected intraperitoneally at the therapeutic doses of 75 mg/Kg, selected from previous toxicity studies, or at vehicle solution. MBoIN179 was dissolved in 10% DMSO in PBS. DMSO toxicity was previously evaluated. Animals were sacrificed 21 days later. Metastasis to the lungs was analyzed by explanting organs, plating dissociated cells in medium supplemented with 6-thioguanine (6-TG), and counting the number of 6-TG-resistant clonogenic tumour cells 2 weeks later.

### Inmunohistochemistry

Indirect immunoperoxidase immunostaining was performed on 4% PFA-fixed paraffin-embedded tumour samples by an automated immunostaining platform (Leica Microsystems Bond RXm). Sections were incubated with the primary antibody Ki67 (D3B5) (#9129, Cell Signaling), Vimentin (ab92546; Abcam) or E-cadherin (24E10) (#3195, Cell Signaling) at 1:400 and then with the corresponding secondary antibody. Slides were counterstained with hematoxylin, dehydrated, and coverslipped. Images were scanned in Leica Aperio Versa at 20x magnification and analyzed with Aperio Image Scope software. Quantification of antibody signal was performed by evaluating the staining of 3-5 fields of 4 different tumours in each condition.

### Immunofluorescence

Cells were fixed with 4% paraformaldehyde, permeabilized with 0.1% Triton X-100 and blocked in 5% BSA in PBS. Cells were labeled with a primary antibody against caspase-3 (9662; Cell Signaling) or against BOK (ab233072; Abcam), followed by anti-mouse or anti-rabbit IgG conjugated to FITC (Jackson ImmunoResearch). Images were obtained using a Leica DMI8 microscope with a 20× objective. Quantification of caspase-3 or BOK signal was performed with ImageJ software analysis, evaluating the ratio between the mean fluorescence value of green signal and DAPI signal in ten different fields from three different tumours for each condition.

### Statistical analysis

Statistical analyses were performed using GraphPad Prism 8 software. Significance was determined by Student’s t-test, one-way ANOVA (followed by Dunnett’s or Tukey’s post-hoc tests), or two-way ANOVA with Sidak’s post-test. A *p*-value < 0.05 was considered statistically significant.

## Supplementary information

Supplemental Material

Original Data

## Data Availability

Molecular dynamics simulation data and parameters have been deposited in Zenodo and are publicly available at the following address: https://doi.org/10.5281/zenodo.17651760. The datasets generated and/or analyzed during the current study are available from the corresponding author on reasonable request.
