# Supplementary material for: Disruption of MCL1/BOK transmembrane interaction as a novel strategy to induce cell death in tumours
Source: Cell Death Dis. 2026 Jul 3;17(1):824. doi: 10.1038/s41419-026-09074-5 (PMC13598098; doi:10.1038/s41419-026-09074-5)
Supplement: Supplementary file 1 [file 41419_2026_9074_MOESM1_ESM.pdf]

Disruption of MCL1/BOK interaction as a novel strategy to induce cell death in tumours.

Buffa M[1,2] *, Leiva D[1] *, A. Garcia-Jareño[1], Garcés-Flores M[1], Díaz E[3,4], Lucantoni F[1], Telechea M[1], Lu Chen[1,5], Lucendo E[1], Shalaby R[6,7], Lolicato F[8,9], Kulig W[9], Garcia-Saez AJ [6,7], Moreno-Bueno G[3,4,10], Sancho M[1,11#] and Orzáez M[1#].

Supplementary Information:

Supplementary Figure Legends

Figure S1.

Figure S2.

Figure S3.

Supplementary Figures

Figure S1.

Figure S2.

Figure S3.

Supplementary Table S1

Supplementary Figure Legends

Figure S1. Overview of the HTS performed for the identification of MCL1/BOK TMD disruptors. (A) Schematic view of BiFC assay analyzing TMD oligomerization by reconstitution of venus fluorescence from separate N (VN) and C (VC) termini fragments fused to TMD segments. (B) MCL1/BOK TMD hetero-oligomerization measured by BiFC quantification in HCT116 cells. MCL1 G340P mutant was already described to decrease interaction. As a negative control for oligomer formation, we employed the alpha 6 helix of BID (BID TMD)[74]. NT refers to non-transfected cells. Graph represents the mean ± SD, n = 3. Significant differences were detected using one-way ANOVA followed by Dunnett's multiple comparison test, ***$P < 0.01$. (C) Chimeric protein expression of VN (myc) and VC (HA) constructs: HA immunoblotting corresponds to VC-BOK TMD expression and myc blotting to VN-MCL1 TMD. α-tubulin was used as loading control. (D) Workflow of the BiFC-based HTS assay showing cell transfection, chemical treatment, and data acquisition steps. (E) Overview of the primary screening data. Each spot represents the percentage of BiFC inhibition of a compound (50 µM) relative to the DMSO-positive control (VN MCL1 TMD / VC BOK TMD). Compounds with at least 60% interaction inhibition were selected out as primary hits. (F-H) Overview of the secondary screening data. Purple circle corresponds to MBoIN179. (F) Representation of compounds with 60% inhibitory activity of MCL1/BOK TMDs interaction. (G) Representation of primary hit inhibition on GpA homo-oligomerization. Compounds with 60% inhibitory activity were excluded. (H) Representation of primary hit inhibition on Venus reconstitution. Compounds with 60% inhibitory activity were excluded. (I) ^{1}H NMR spectra of compound MBoIN179 in deuterated DMSO (300 MHz). (J-K) Quantification by densitometry of the three western blot replicates for protein expression level of the BiFC constructs from Figure 1C and E, respectively. Data were analyzed using one-way ANOVA followed by Tukey's post-hoc test. (L) Homo-oligomerisation analysis of BOK TMD constructs measured by BiFC in HCC1954 cells in the presence of MBoIN179 (27 µM). The left panel illustrates the BiFC signal upon MBoIN179 treatment. Graph represents the mean ± SD, n = 3. Data were analyzed using one-way ANOVA followed by Tukey's post-hoc test; **$p < 0.01$. Protein expression level of the constructs and quantification are shown in the right panels. (M) Illustration of the BOK and (N) MCL-1 binding to MBolN179.

Figure S2. (A) Quantification by densitometry of the three western blot replicates for protein expression level of the BiFC constructs from Figure 2J. Data were analyzed using one-way ANOVA followed by Tukey's post-hoc test. (B) Quantification by densitometry of the three western blot replicates for protein expression level of the BiFC constructs from Figure 3H. Data were analyzed using one-way ANOVA followed by Tukey's post-hoc test. (C) Flow cytometry analysis of

HCC1954 cells stained with Annexin V after treatment with MBolN179 (27 µM) for 48h. The percentage of Annexin V–positive cells was determined from 10,000 events analyzed per condition. STS refers to staurosporine treatment (100 nM) as a positive control of apoptosis induction. (D) Quantification by densitometry of the three western blot replicates for protein expression level of the BiFC constructs from Figure 4C. (E) Flow cytometry analysis of 4T1 cells stained with Annexin V after treatment with MBolN179 (40 µM) for 24h. The percentage of Annexin V–positive cells was determined from 10,000 events analyzed per condition. STS refers to staurosporine treatment (1 µM) as positive control of apoptosis induction. (F) Caspase 3/7 activity induced by MBoIN179 (27 µM) for 48h in HCC1954 control and overexpressing BOK in a BOK silenced context. Graph represents the mean ± SD, of n = 3. Data were analyzed using one-way ANOVA followed by Dunnett's post-hoc test; *p < 0.05. Immunoblot panel shows BOK expression in graph, α-tubulin is used as loading control. (G) Caspase 3/7 activity in HCC1954 control (mock) and overexpressing MCL1 or MCL1DTM after 48h of MBoIN179 (27 µM) treatment. Graph represents the mean ± SD, of n = 3. Data were analyzed using one-way ANOVA followed by Dunnett's post-hoc test; *p < 0.05. Immunoblot panel shows MCL1 constructs expression; α-tubulin is used as loading control. (H) Kinetic analysis of calcein release from LUVs in the presence of MBoIN179 (50 nM) and BOK (125 nM).

Figure S3. Toxicity evaluation of 4T1-TNBC mice treated with MBoIN179. (A) Red blood cells, white blood cells, haemoglobin level and platelet numbers in blood of 4T1-TNBC mice after treatment with vehicle or MBoIN179 compound (50 mg/ml). (B) Detailed characterization of subpopulations of white blood cells in these animals. Graphs for neutrophile, lymphocyte and monocyte content are shown. Almost no detection was observed in basophile and eosinophile population. (C) Liver, heart and muscle damage was evaluated by detecting changes in creatine phosphokinase (CPK), aspartate aminotransferase (AST/GOT), glutamate pyruvate aminotransferase (GPT) and lactate dehydrogenase (LDH) activity. (D) Survival rate of mice treated with vehicle or MBoIN179 along 14 days. (E) Follow-up of the weight of animals treated with vehicle or MBoIN179 during the study. (F) Representative haematoxylin and eosin (H&E)-stained sections of heart tissue from mice after the end of treatment with either vehicle or MBoIN179. Scale bars represent 100 µm for the ×40 objective.

Figure S1

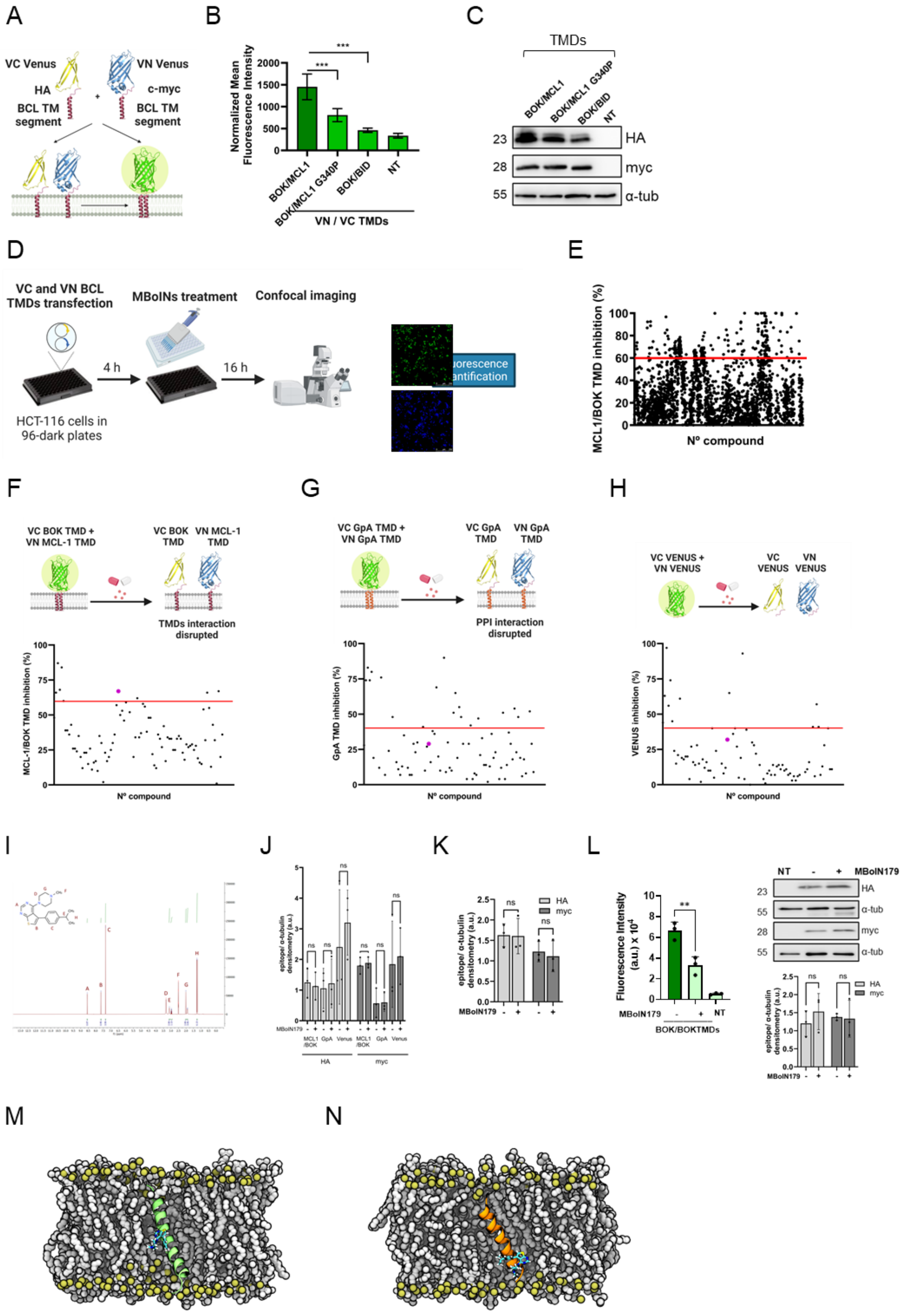
A
VC Venus
VN Venus
HA
c-myc
BCL TM segment
BCL TM segment
B
Normalized Mean Fluorescence Intensity
***
***
BOK/MCL1
BOK/MCL1 G340P
BOK/BID
NT
VN / VC TMDs
C
TMDs
BOK/MCL1
BOK/MCL1 G340P
BOK/BID
NT
23
HA
28
myc
55
α-tub
D
VC and VN BCL TMDs transfection
MBoINs treatment
Confocal imaging
4 h
16 h
HCT-116 cells in 96-dark plates
E
MCL1/BOK TMD inhibition (%)
Nº compound
F
VC BOK TMD + VN MCL-1 TMD
VC BOK TMD
VN MCL-1 TMD
TMDs interaction disrupted
MCL-1/BOK TMD inhibition (%)
Nº compound
G
VC GpA TMD + VN GpA TMD
VC GpA TMD
VN GpA TMD
PPI interaction disrupted
GpA TMD inhibition (%)
Nº compound
H
VC VENUS + VN VENUS
VC VENUS
VN VENUS
VENUS inhibition (%)
Nº compound
I
J
K
HA
myc
MBoIN179
L
Fluorescence Intensity (a.u.) x 10^4
**
MBoIN179
NT
BOK/BOKTMDs
HA
α-tub
myc
MBoIN179
M
N

Figure S2

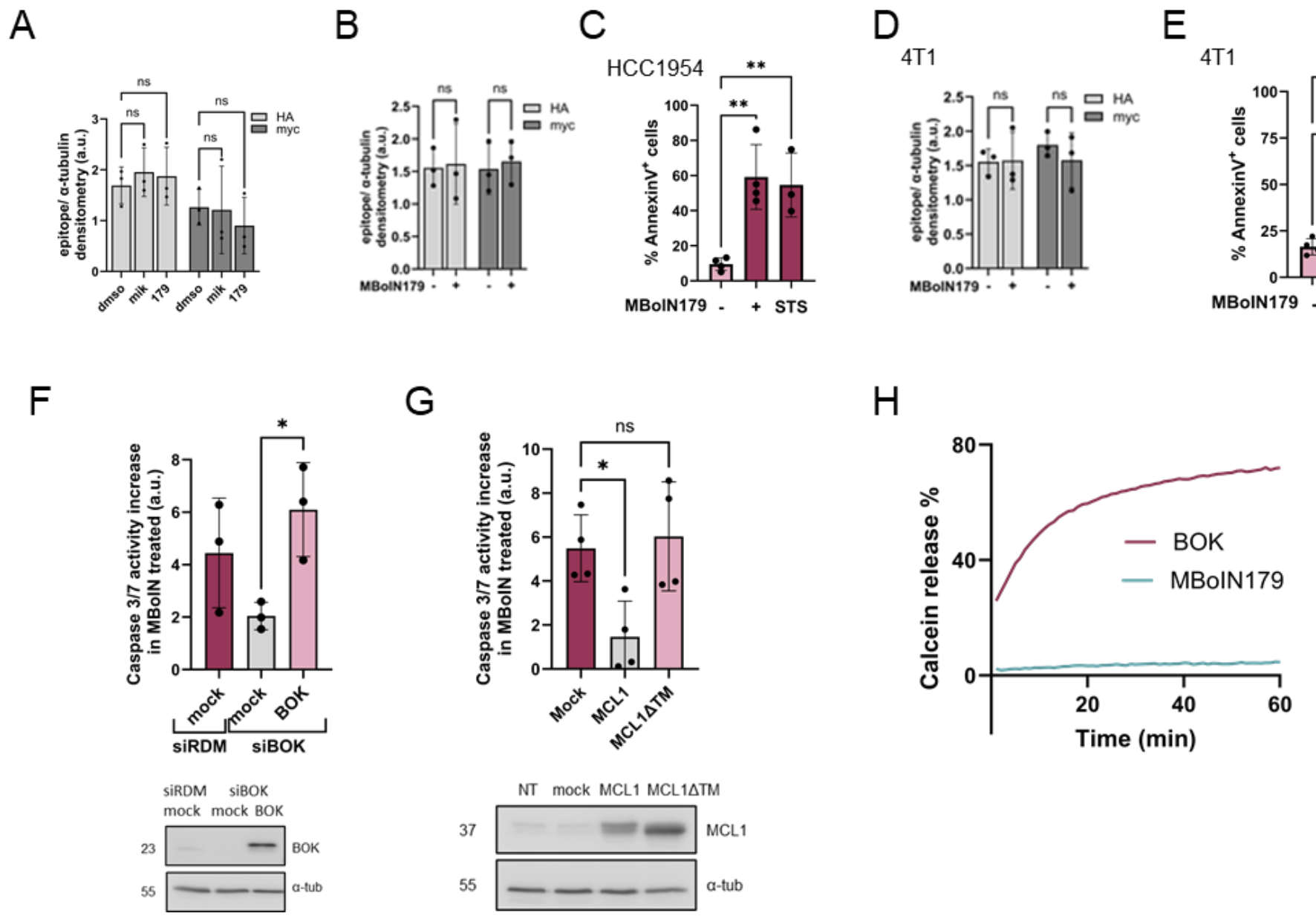
A
B
C
D
E
HCC1954
4T1
4T1
F
G
H
HA
myc
ns
epitope/ α-tubulin densitometry (a.u.)
dmso
mik
179
MBoIN179
% AnnexinV+ cells
STS
Caspase 3/7 activity increase in MBoIN treated (a.u.)
mock
BOK
siRDM
siBOK
Mock
MCL1
MCL1ΔTM
NT
α-tub
Calcein release %
MBoIN179
Time (min)

Figure S3

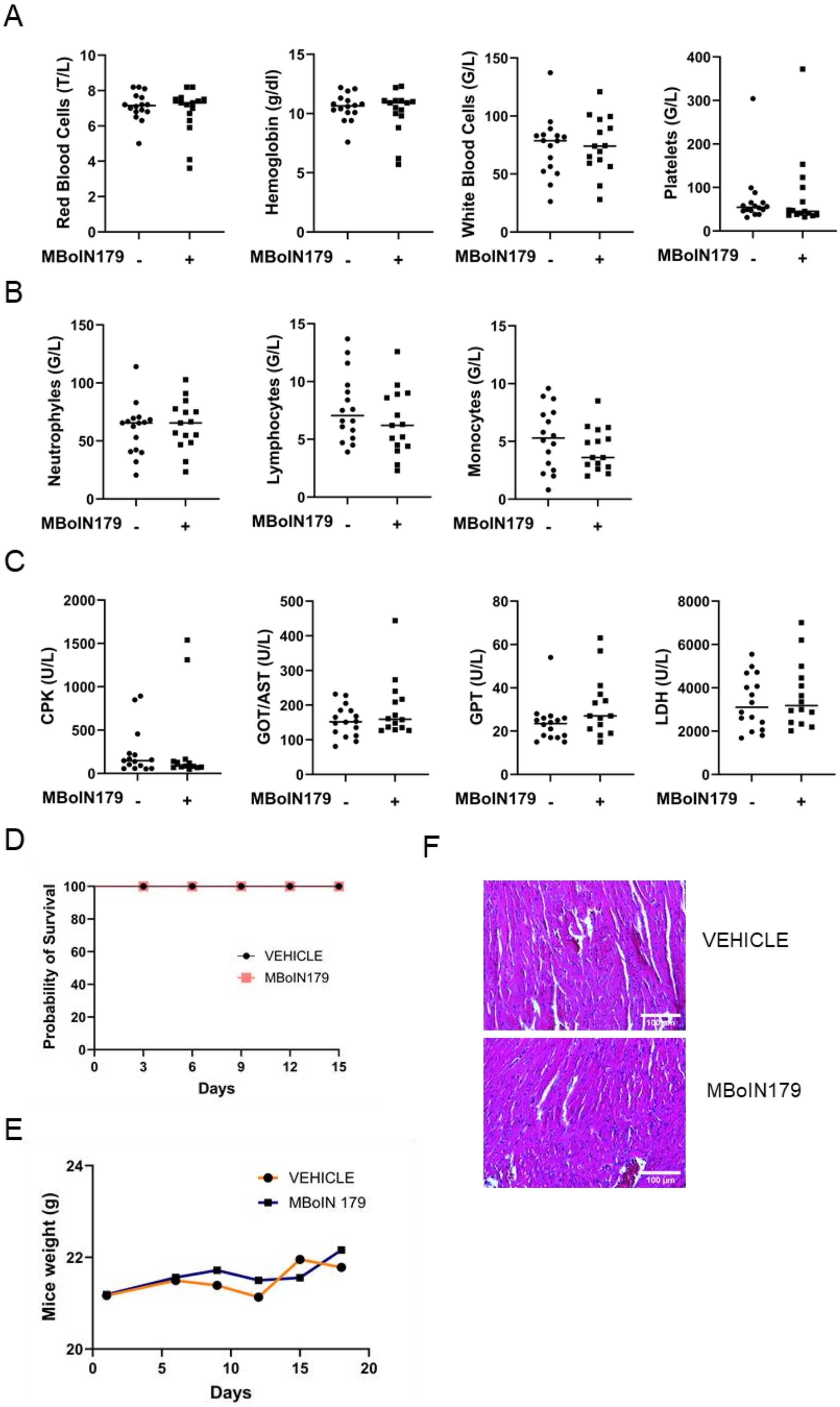

A
Red Blood Cells (T/L)
Hemoglobin (g/dl)
White Blood Cells (G/L)
Platelets (G/L)
MBoIN179 - +
B
Neutrophyles (G/L)
Lymphocytes (G/L)
Monocytes (G/L)
MBoIN179 - +
C
CPK (U/L)
GOT/AST (U/L)
GPT (U/L)
LDH (U/L)
MBoIN179 - +
D
Probability of Survival
Days
VEHICLE
MBoIN179
E
Mice weight (g)
Days
VEHICLE
MBoIN 179
F
VEHICLE
MBoIN179
100 µm

Table S1. Summary of clinical, pathological, immunohistochemical and molecular features variables in the cohort of 207 samples.

	n (%)
**Tumor grade (n=186)**	
1	17 (9%)
2	71 (38%)
3	98 (53%)
**Molecular Subtype (n=202)**	
Luminal A	115 (57%)
Luminal B	41 (20%)
HER2	15 (7%)
TNBC	31 (15%)
**Lymph node metastasis (n=196)**	
No	128 (65%)
Yes	68 (35%)
**Distant metastasis (n=204)**	
No	171 (84%)
Yes	33 (16%)
**Estrogen Receptor (n=201)**	
Negative	48 (24%)
Positive	153 (76%)
**Progesterone Receptor (n=200)**	
Negative	65 (32%)
Positive	135 (68%)
**HER2 (n=196)**	
Negative	152 (78%)
Positive	44 (22%)
**BOK Expression (n=128)**	
Negative	95 (74%)
Positive	33 (26%)
