# Supplementary material for: Disruption of MCL1/BOK transmembrane interaction as a novel strategy to induce cell death in tumours
Source: Cell Death Dis. 2026 Jul 3;17(1):824. doi: 10.1038/s41419-026-09074-5 (PMC13598098; doi:10.1038/s41419-026-09074-5)

Figure 1

C

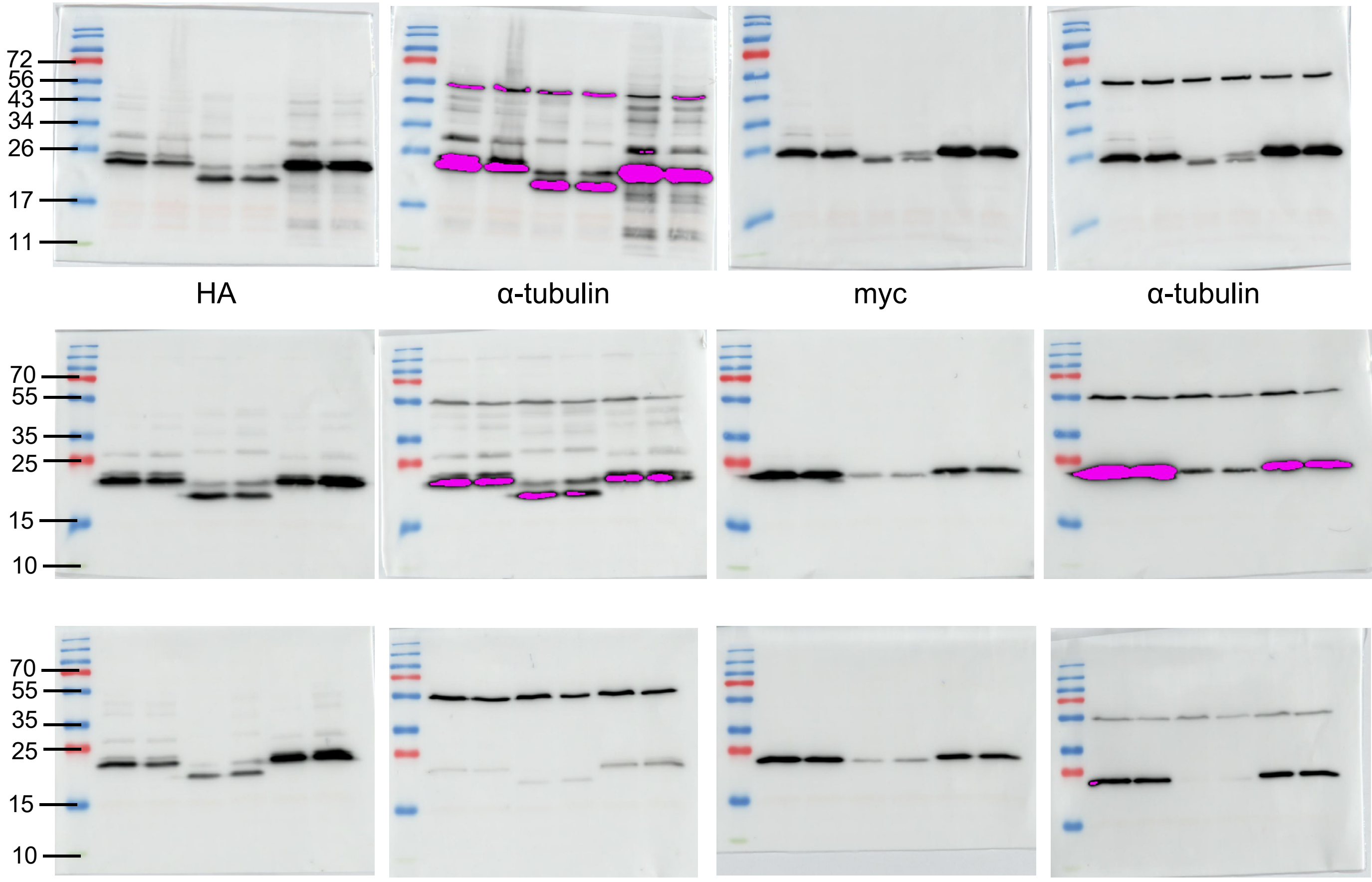
72
56
43
34
26
17
11
HA
α-tubulin
myc
α-tubulin
70
55
35
25
15
10
70
55
35
25
15
10

E

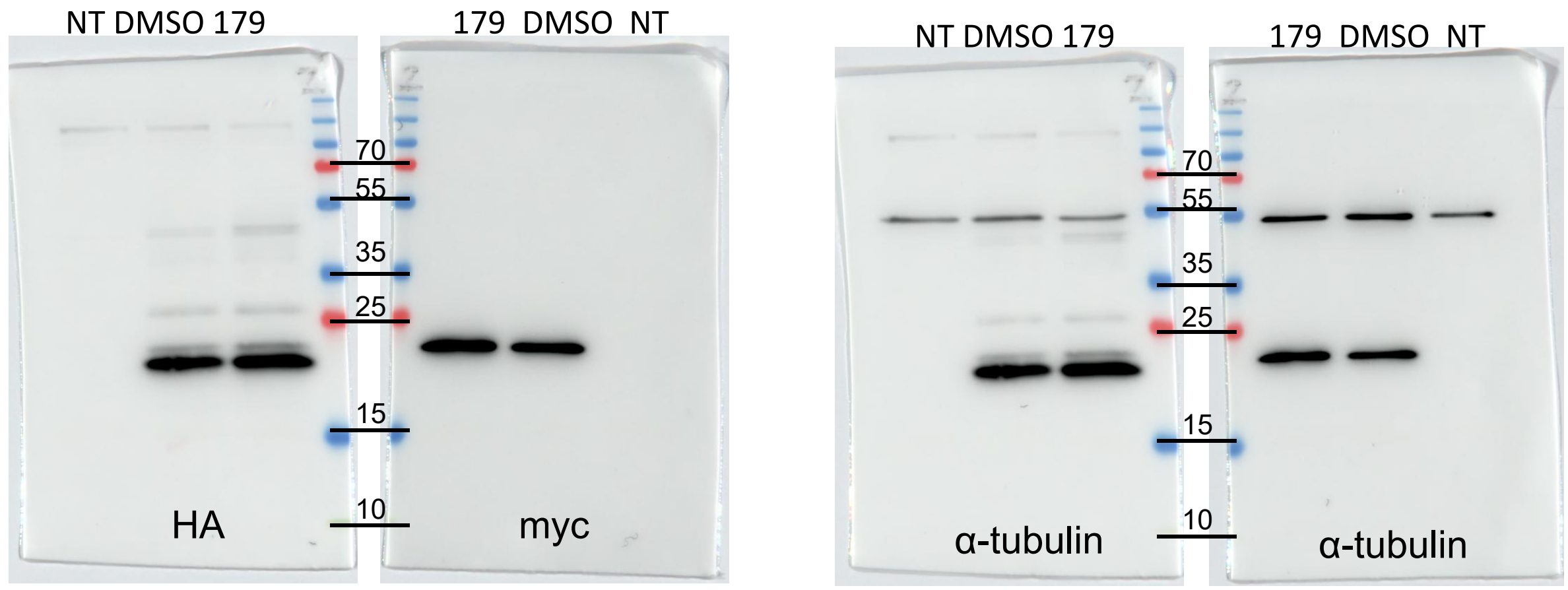
NT DMSO 179
179 DMSO NT
70
55
35
25
15
10
HA
myc
NT DMSO 179
179 DMSO NT
70
55
35
25
15
10
α-tubulin
α-tubulin

Figure 1

L

Mitochondria

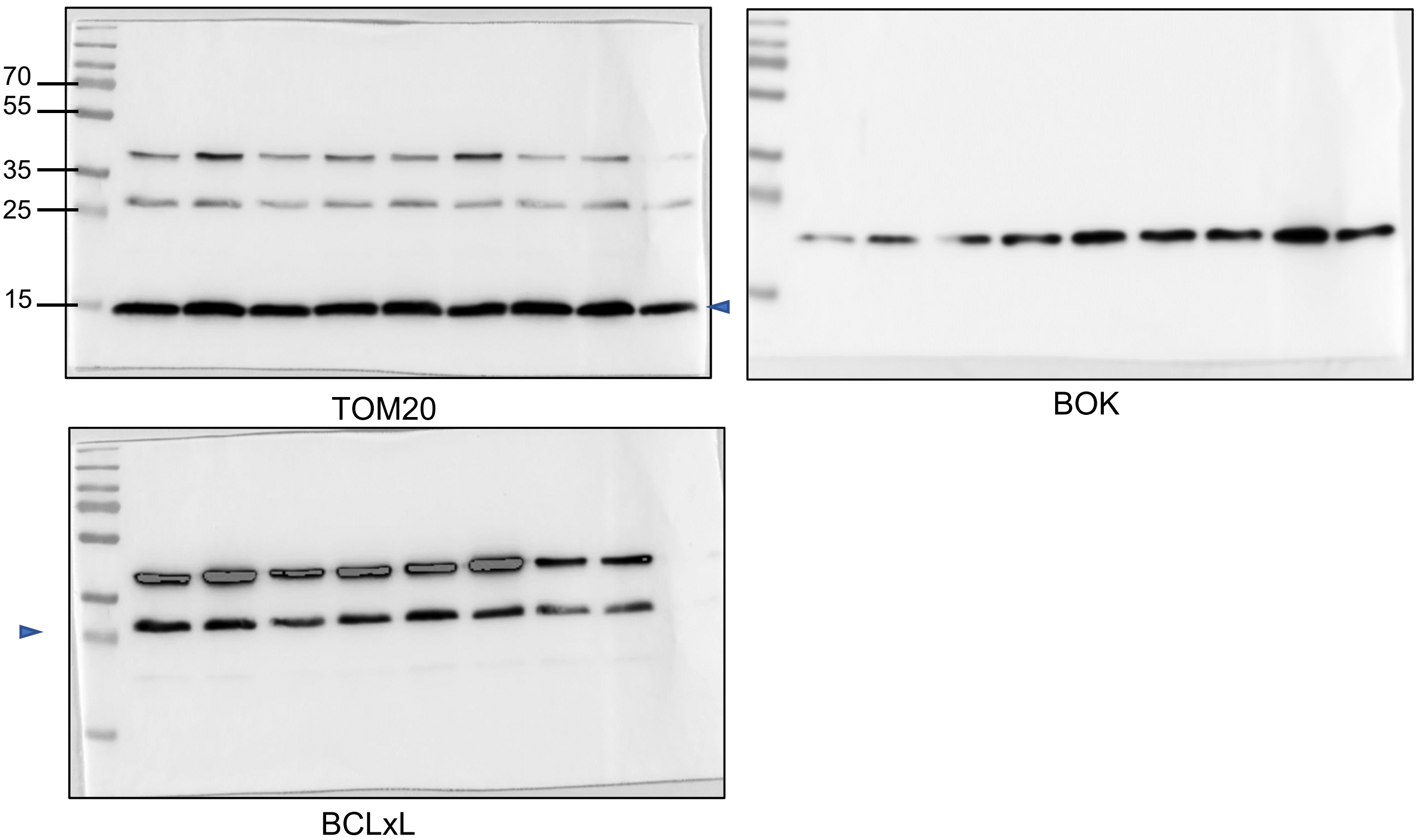

Whole cell extract

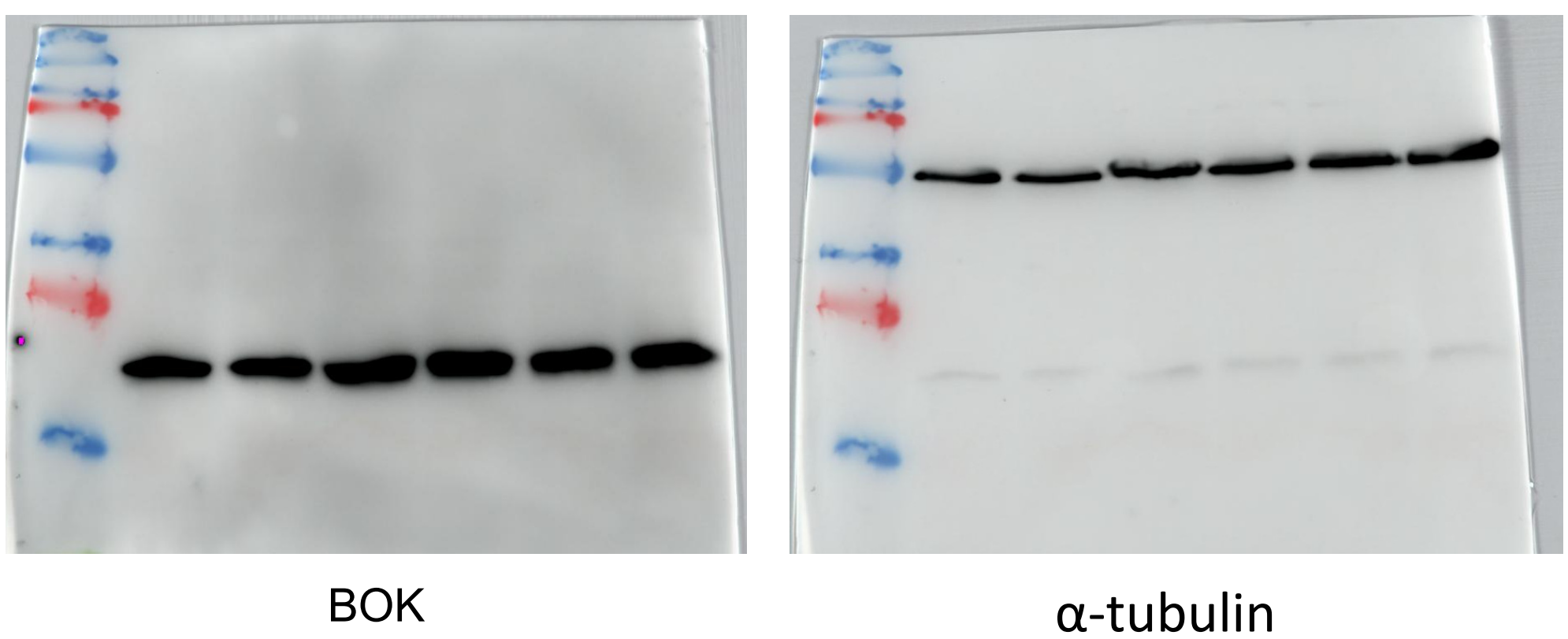

Figure 2

B

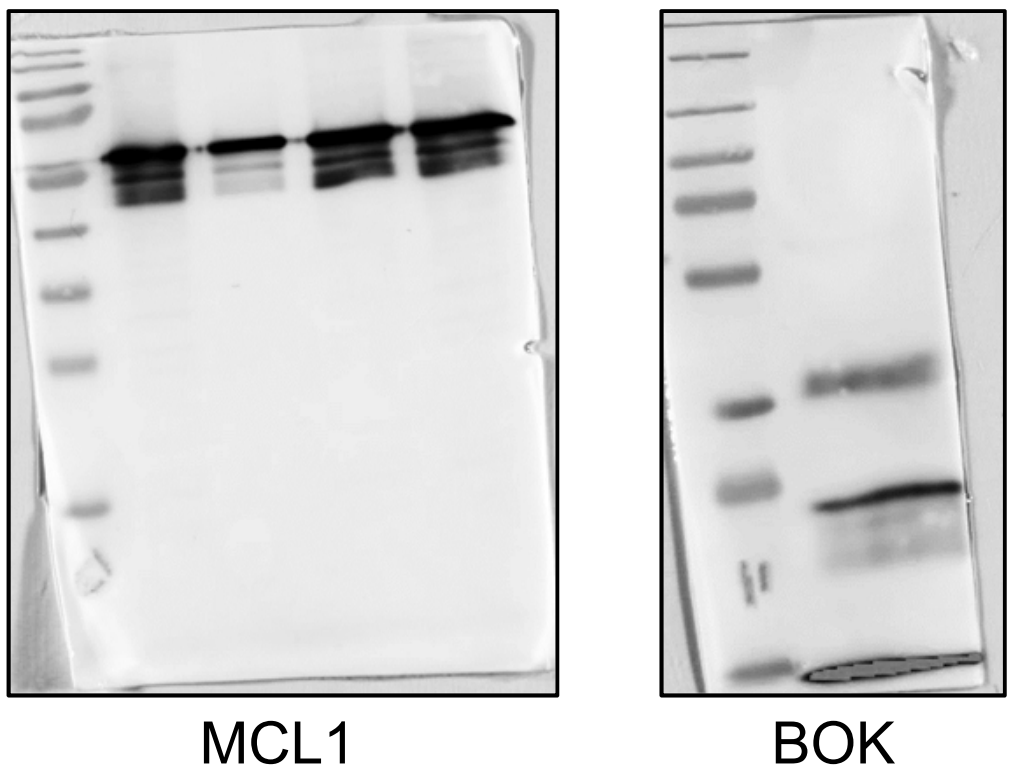

MCL1
BOK

J

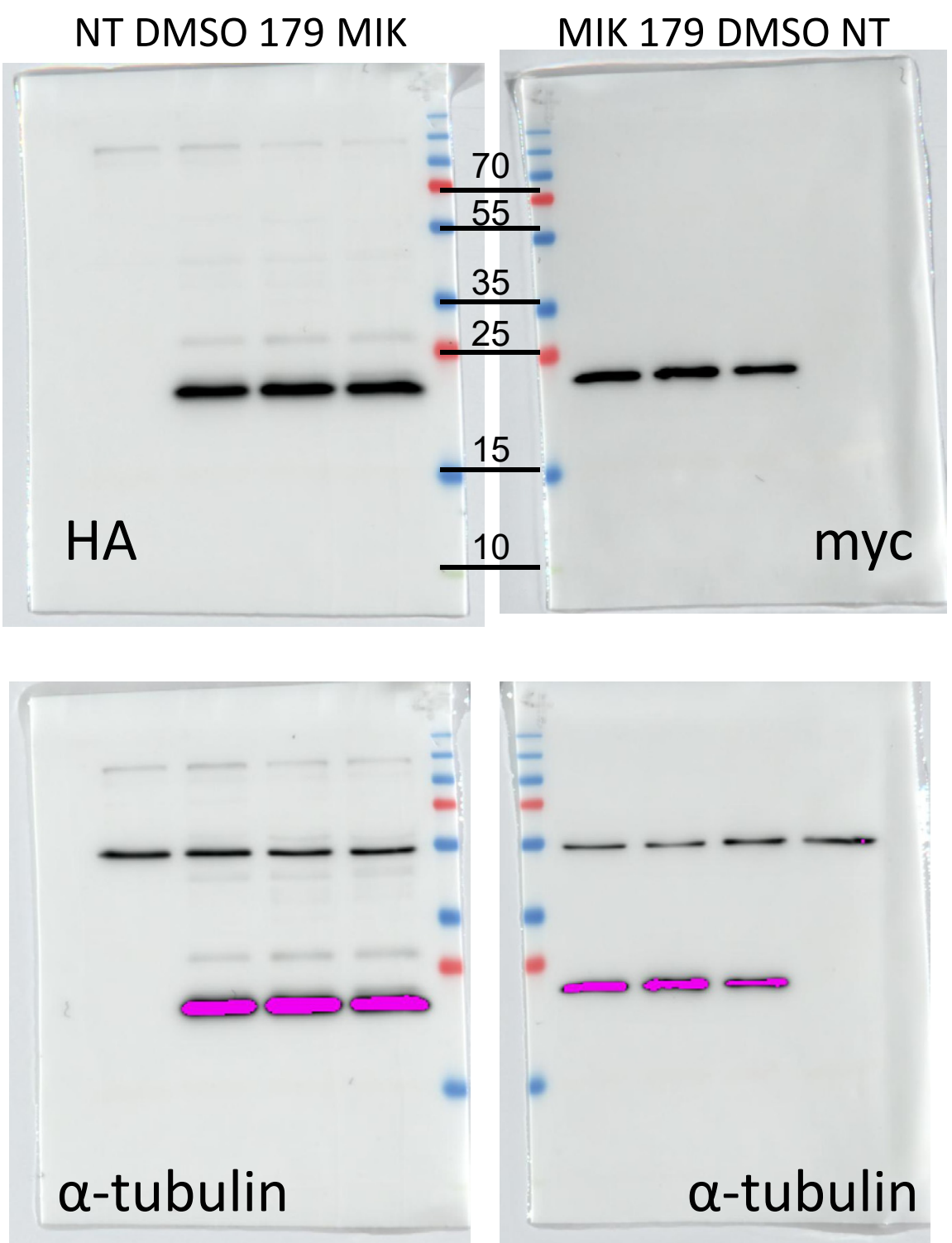

NT DMSO 179 MIK
MIK 179 DMSO NT
70
55
35
25
15
10
HA
myc
α-tubulin
α-tubulin

Figure 3

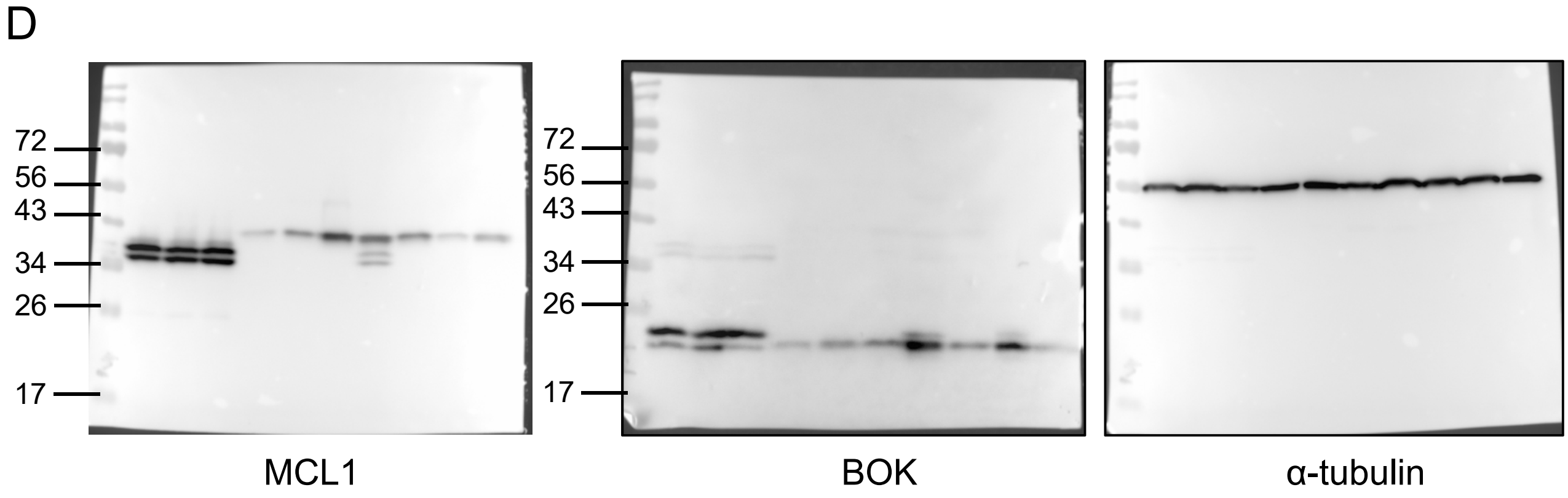
D
72
56
43
34
26
17
MCL1
BOK
α-tubulin

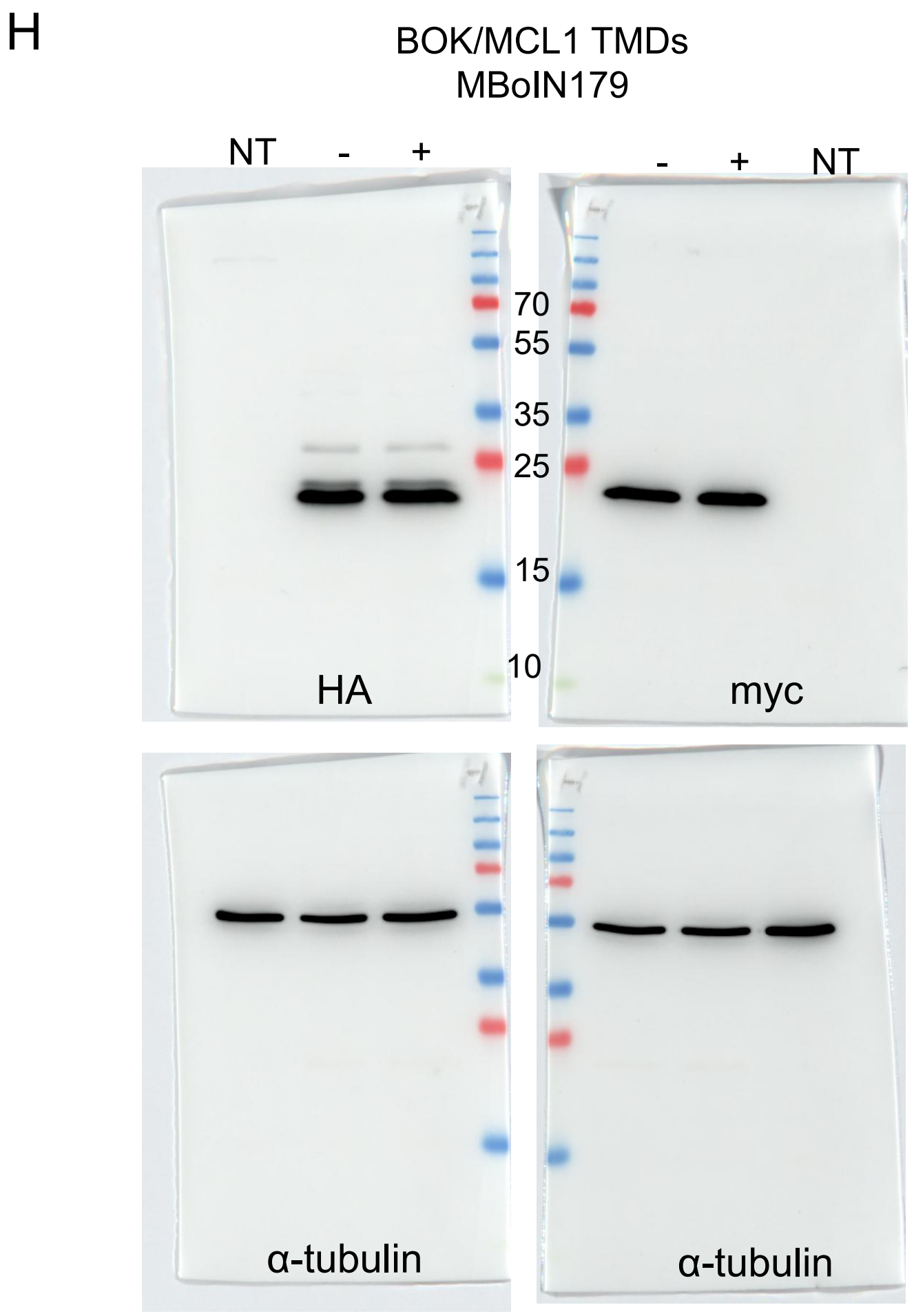
H
BOK/MCL1 TMDs
MBoIN179
NT - +
- + NT
70
55
35
25
15
10
HA
myc
α-tubulin
α-tubulin

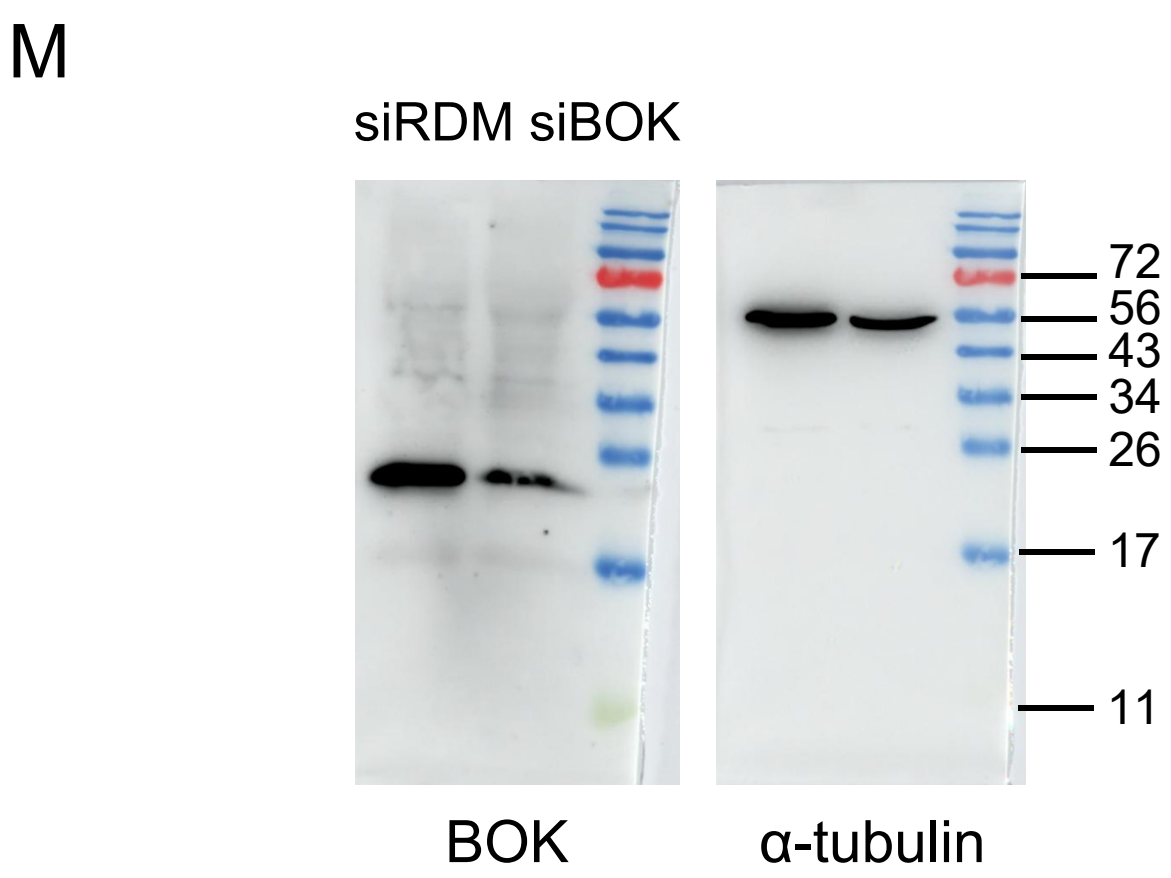
M
siRDM siBOK
72
56
43
34
26
17
11
BOK
α-tubulin

Figure 4

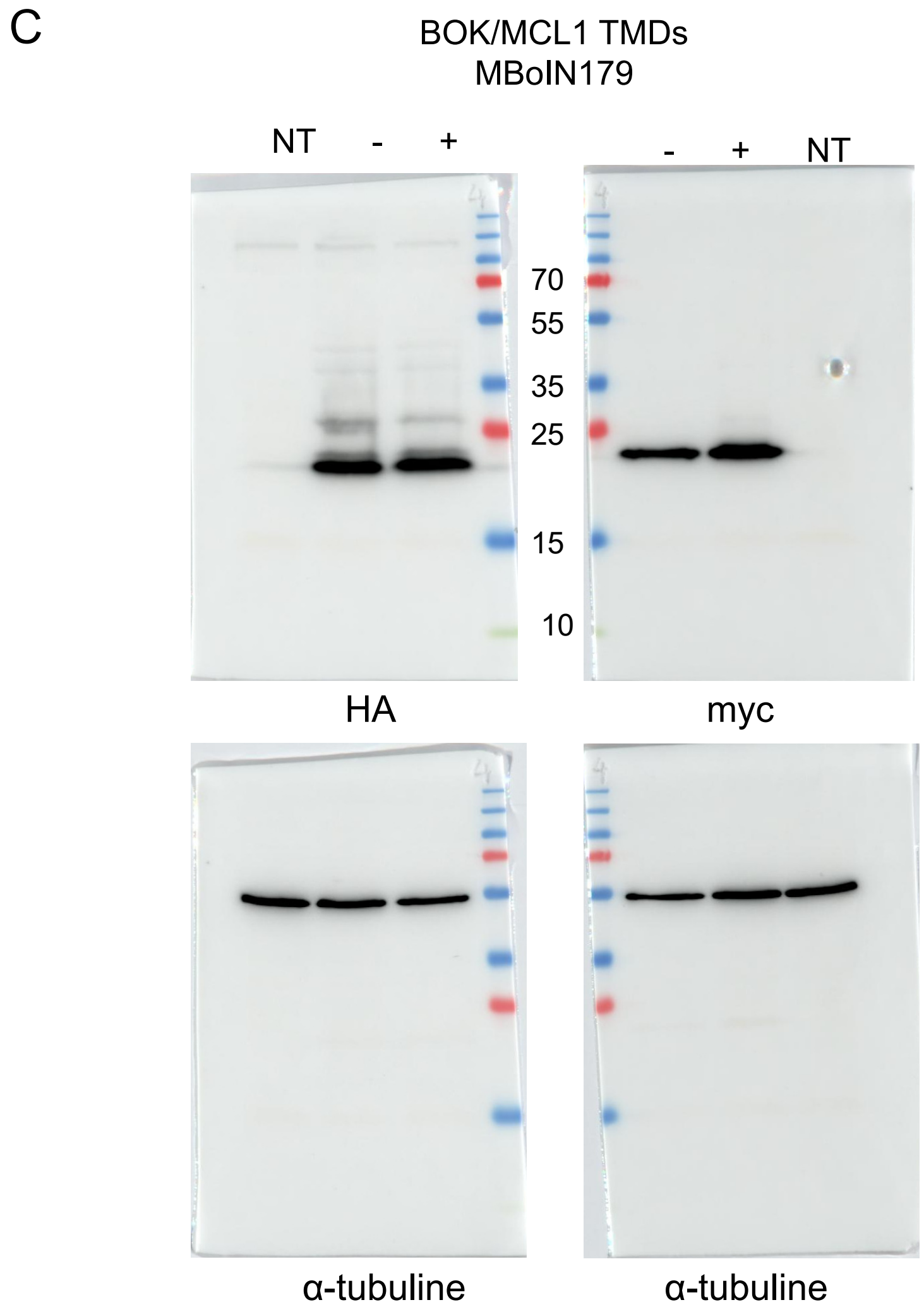
C
BOK/MCL1 TMDs
MBoIN179
NT
-
+
-
+
NT
70
55
35
25
15
10
HA
myc
α-tubuline
α-tubuline

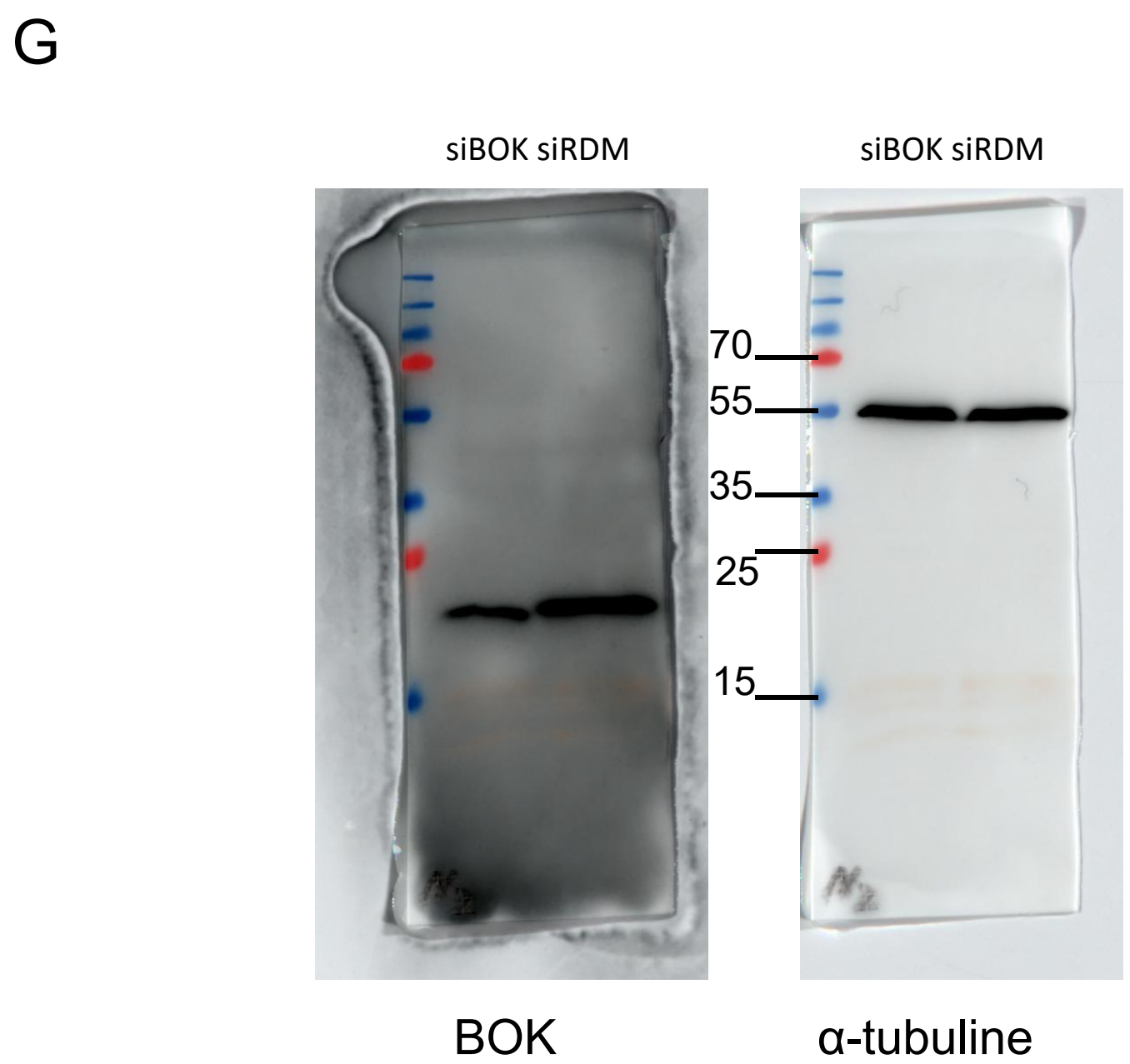
G
siBOK siRDM
siBOK siRDM
70
55
35
25
15
BOK
α-tubuline

Figure S1

L

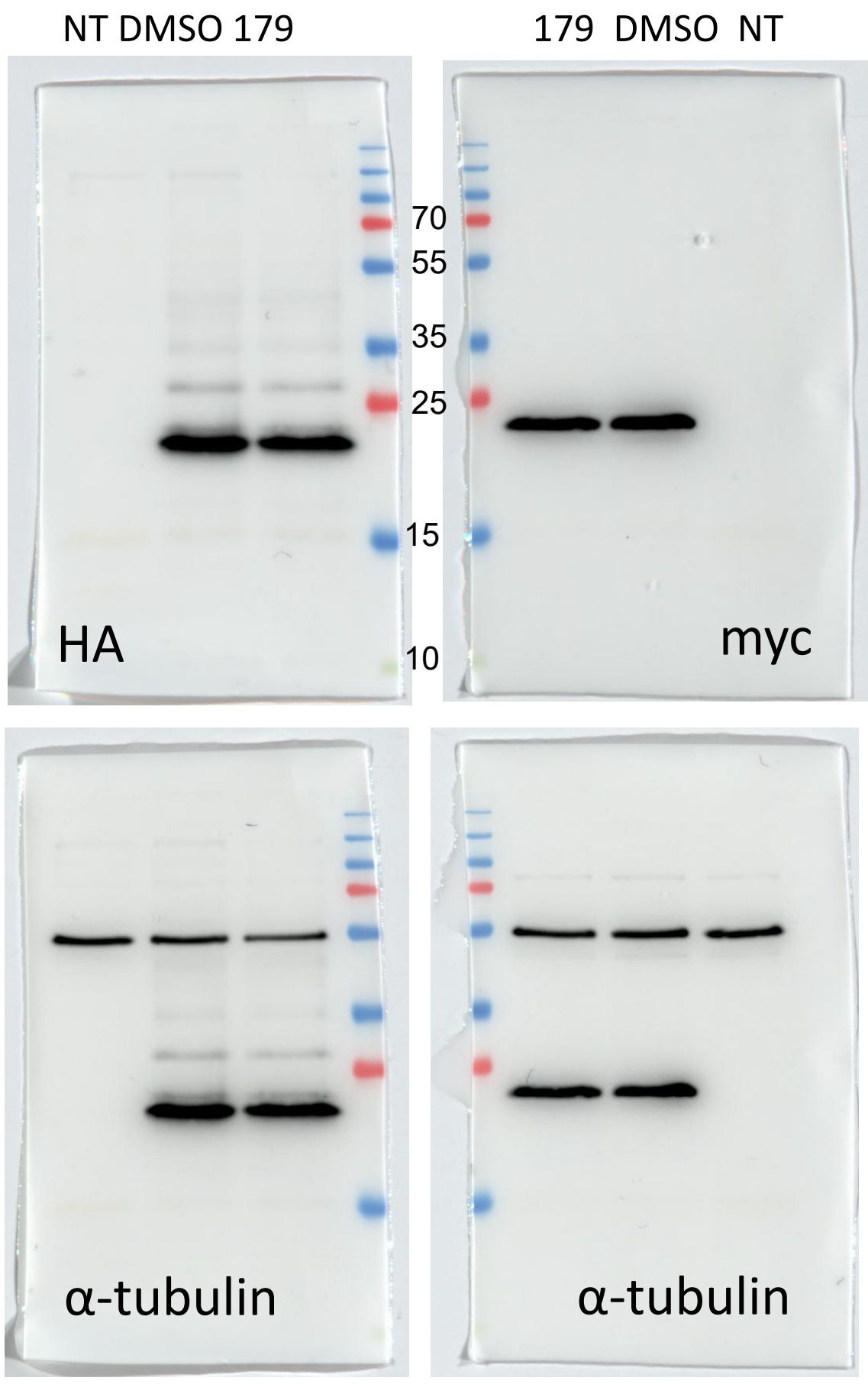

Figure S2

F

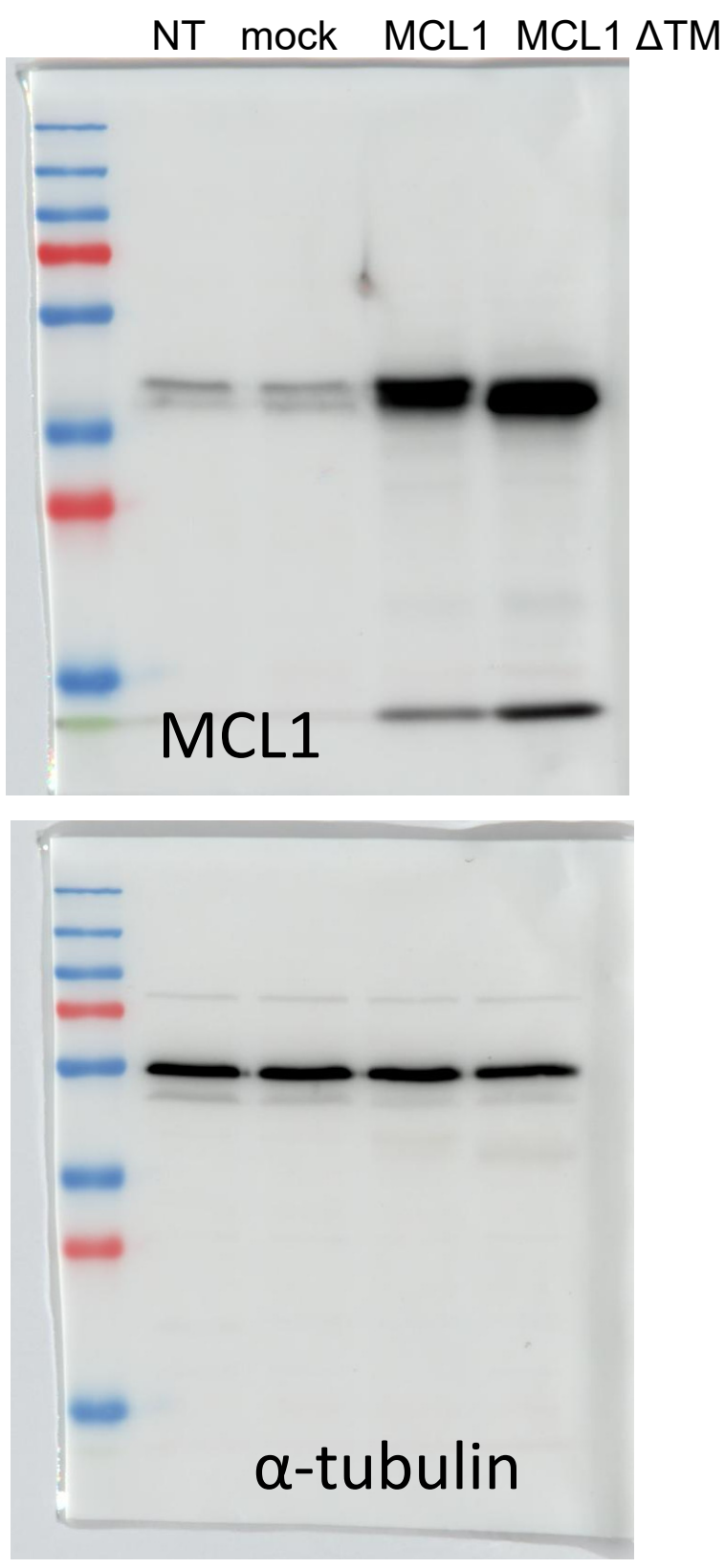

G

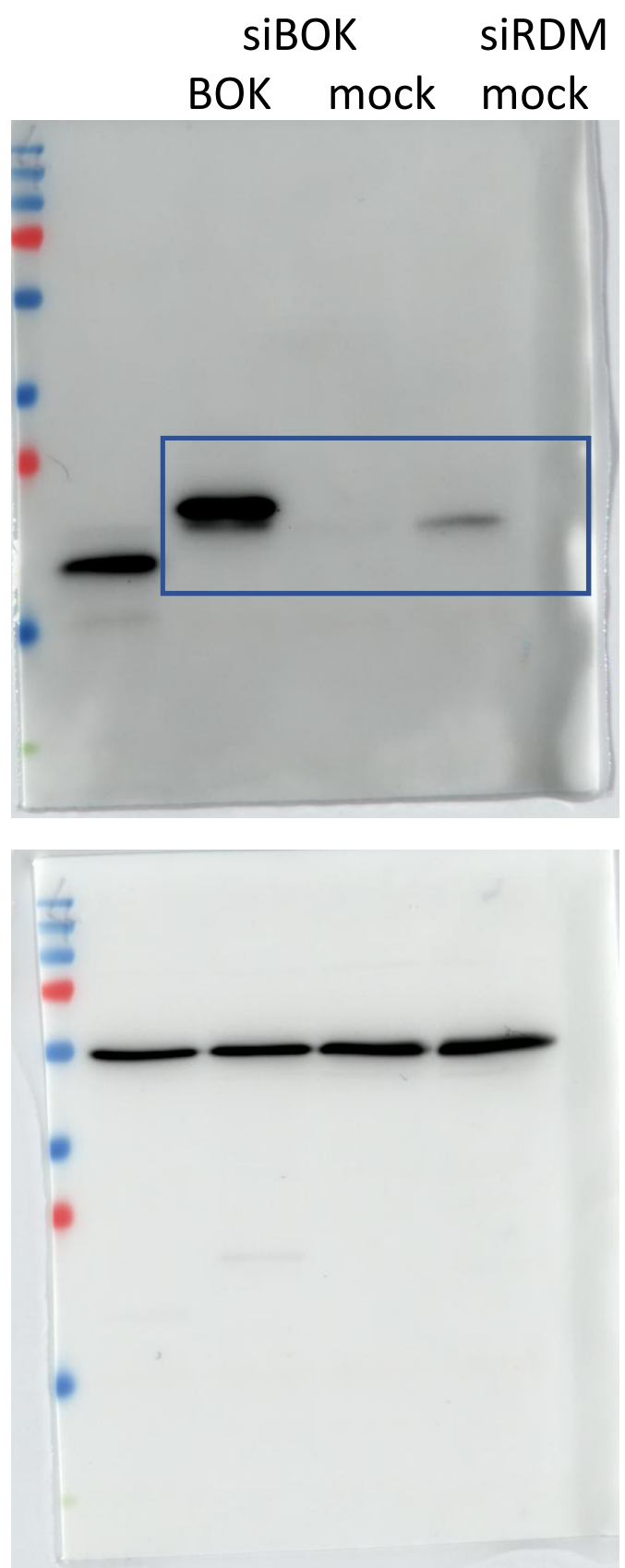

Supplement: Supplementary file 2 [file 41419_2026_9074_MOESM2_ESM.pdf]
